# Synthesis, computational, biological activity and molecular docking study of Co^2+^, Ni^2+^ and Cu^2+^ chelates of a new indolbenzohydrazone NO donor

**DOI:** 10.1038/s41598-025-20172-w

**Published:** 2025-10-28

**Authors:** Rashy E. Georgy, Mahmoud N. Abd El-Hady, Ola A. El-Gammal

**Affiliations:** Chemistry Department, Faculty of Science, Mansoura University, P.O. Box 35516, Mansoura, Egypt

**Keywords:** Schiff base, Antibacterial, Free radicals scavenging, Breast cancer, Molecular docking, Biochemistry, Cancer, Chemistry

## Abstract

A novel Schiff base namely, (*Z*)-4-((3-cyano-4,6-dimethylpyridin-2-yl)amino)-*N*′-(2-oxoindolin-3-ylidene)benzohydrazide (H_2_BISH) (C1) and its corresponding metal acetate chelates; [Co_2_(HBISH)(CH_3_COO)_3_(H_2_O)_2_]·H_2_O (C2), [Ni(HBISH)(CH_3_COO)]·2.5H_2_O (C3) and [Cu(HBISH)(CH_3_COO)(H_2_O)] (C4) were prepared and characterized using elemental and spectroscopic (IR, UV–vis., nuclear magnetic resonance (^1^H&^13^C), electron paramagnetic resonance (ESR), mass and XRD measurements. IR spectral data revealed that H_2_BISH acts as monobasic in all metal complexes. The ^1^HNMR chemical shifts of the ligand protons are relatively well reproduced at the HF/6-31G(d) level of theory. Ni^2+^ complex (C1) adopted a tetrahedral structure while Co^2+^ (C2) and Cu^2+^ complexes assigned an octahedral one. The structure of C2 was further investigated by XRD technique orthorhombic C_30_ Co_3_ N_22_O_5_ geometry with lattice constants a = 6.5688 Å, b = 31.7110 Å and c = 18.1864 Å and Cm c m space group. Kinetic parameters of thermal degradation of studied compounds were evaluated by Coats–Redfern and Horowitz–Metzger equations. Density function theory was utilized to draw the structures and evaluate the correlated parameters. All title compounds have small negative E_g_ of order: C2 < C3 < C4 < C1 (H_2_BISH) revealing the ease of CT in turn the higher polarizability, increased reactivity and softness as well as the values lie in the range of semiconductor suggesting the possibility of utilization of present compounds in solar cells. The compounds were tested for antioxidant power (DPPH free radical scavenger assay) and bacterial inhibition against *Bacillus cereus* as Gram + ve and *Escherichia coli* as Gram − ve bacteria. A molecular docking mechanism between the ligand and its M^2+^ chelating was evaluated against bacterial proteins and Breast cancer cells sites receptors to explain how these compounds attach the protein’s active sites. The findings showed promising binding.

**Supplementary Information:**

The online version contains supplementary material available at 10.1038/s41598-025-20172-w.

## Introduction

Nowadays, the formation of complexes of active compounds especially hydrazones using metal ions as coordinate centers is one of the methods which can enhance and improve their properties as active molecules. Also, it is possible to manipulate the compound’s activity by changing the metal ion in the complex^1^. Hydrazones contain the imine group –C=N formed by condensation of ketone or aldehyde with RNHNH_2_ still attract attention owing to their coordination diversity and wide spread pharmaceutical and industrial applications. The presence of such azomethine linkages distinguishes hydrazonoic chelators from other Schiff bases, which makes them particularly desirable building blocks for the construction of bioactive transition metal complexes^2–4^. Also, hydrazonic links cause significant effects on the chemical and physical properties due to the electrophilic character of nitrogen atoms and the nucleophilic and electrophilic characteristics of carbon atoms which enabled scientists to design significant hydrazonic chelator derivatives for medicinal and pharmaceutical purposes by altering the substitution groups^5–7^. The hydrazone compounds showed an important activity in these purposes including antimicrobial as antibacterial and antifungal^8–10^, enzyme inhibitors^11^, anti-trypanosome^12^, anti-inflammatory^13^ antiviral^14^, antitumor^15^, anticancer^16^, antidiabetic^17^ and antioxidant^18^. Additionally, several acyl-hydrazone derivatives have been used in the inhibition of myeloperoxidase/acetylcholinesterase, radical scavenging, and treatment of Alzheimer’s disease^19^. Hydrazones in particular those derived from isatine ketonic derivative found a great attention in coordination chemistry as isatin, either by itself or in combination with other ligands, possesses the potential to be a good substrate for the synthesis of metal complexes because of its cis α-dicarbonyl moiety carrying an extra heteroaromatic ring provides a powerful ligand. The isatin moiety behaves as a potential ambidentate ligand that can bind the metal ions in enol or keto modes^20,21^. We have reported the synthesis and in vitro antibacterial and antitumor on human mammary gland (breast) MCF7 activities of picolinohydrazide derivative: (*Z*)-N0-(2-oxoindolin-3-ylidene)picolinohydrazide (H_2_IPH) and its Co(II), Ni(II) and Cu(II) complexes on human mammary gland (breast) MCF7^22^. Chelation in the context of enzyme active sites refers to the process where a molecule, called a chelating agent, binds to a metal ion within the enzyme’s active site, creating a specific geometric arrangement. This interaction can affect the enzyme’s ability to bind to its substrate and catalyze a reaction, effectively “locking” the enzyme into an active or inactive state depending on the specific chelation interaction. Thus, by chelating the active sites, the metal ions can create a particular “lock geometry” that allows certain molecules to connect to the framework and activate the enzymes^23^.

Copper (II), which itself has biological activity such as blood–brain barrier penetration, proteasome activity inhibitors and virus C inhibitor. Excess of copper in the human organism can cause Alzheimer’s Disease^24^. Also, Cobalt (II) is found in Vitamin B12, an essential nutrient for humans and animals. It plays a crucial role in many enzymatic processes, including those that produce red blood cells, amino acid metabolism, fix nitrogen and transport methyl^25^ whereas Nickel (II) is component of several enzymes, including urease, hydrogenase, and carbon monoxide dehydrogenase, and is involved in various biochemical processes. Some nickel complexes have demonstrated antioxidant, anti-inflammatory, antiallergic and anticancer activities^26,27^. On the basis of these facts and on continuation of work reported, study of the ability of novel (*Z*)-4-((3-cyano-4,6-dimethylpyridin-2-yl)amino)-*N*′-(thiophen-2-ylmethylene)benzohydrazide (H_2_BTH) and its mononuclear Zn(II), Cd(II) and Hg(II) complexes to inhibit the growth of bacterial and fungal strains as well as DNA binding^28^, we describe herein the synthesis, characterization and in vitro antibacterial, antioxidant and breast anticancer activities of a novel Schiff base; (*Z*)-4-((3-cyano-4,6-dimethylpyridin-2-yl)amino)-*N*′-(2-oxoindolin-3-ylidene)benzohydrazide (H_2_BISH) and its Co^2+^, Ni^2+^ and Cu^2+^ acetate chelates. Currently, we are interest in investigation of a series of chelates derived from the investigated carbohydrazide. The recent hydrazone derivative is unique as our research group have studied and prepared a series of such Schiff bases. Also, the structure of the present Schiff base hydrazone contains a heterocycle moiety, viz pyridine carrying a terminal CN group, phenyl and indol hydrazide moiety that are experience a great potential antioxidant power.

## Experimental

### Chemicals and measurements

All the chemicals used were of analytical grade, obtained from Sigma-Aldrich (St. Louis, MO, USA). Infrared spectra were measured using Mattson-5000 FTIR Spectrophotometer (KBr discs, 4000–400 cm^−1^), Carbon, hydrogen and nitrogen percent were determined by standard Methode^28^ using Perkin–Elmer 2400 series II analyzer. Electronic spectra were displayed using Perkin–Elmer UV–visible spectra using the AA800 spectrophotometer Model AAS with a 1.0 cm cell model. NMR spectra in DMSO-d_6_ were shown using Bruker WP operating at 300 and 80 MHz, respectively. TGA & DrTGA was recorded using Shimadzu thermogravimetric analyzer under atmospheric N_2_ at (R.T. − 800 °C) with a heating rate of 15 °C/min and using α-Al_2_O_3_ as a reference.

A powder ESR spectrum was performed using Bruker EMX spectrometer in quartz capillary tube (2 mm) at room temperature the spectrophotometer works in 9.78 GHz X-band (with 100 kHz modification frequency) using DPPH as a standard. The diffractometer (Bruker AXS Advance) with the following features (for Cu(II) the Wavelength equal 1.5406 Å source) was used to record the XRD spectrum. Match computer program was used for measuring and optimizing the values of space group, crystal scheme and lattice parameters of the Co(II) complex. The Spekol 11 spectrophotometer; analytic Jena AG, Jena, Germany, was used for measuring DPPH antioxidant activity.

### Synthesis of (***Z***)-4-((3-cyano-4,6-dimethylpyridin-2-yl)amino)-***N***′-(2-oxoindolin-3-ylidene)benzohydrazide (H_3_BISH) and its metal complexes.

**Scheme 1:**
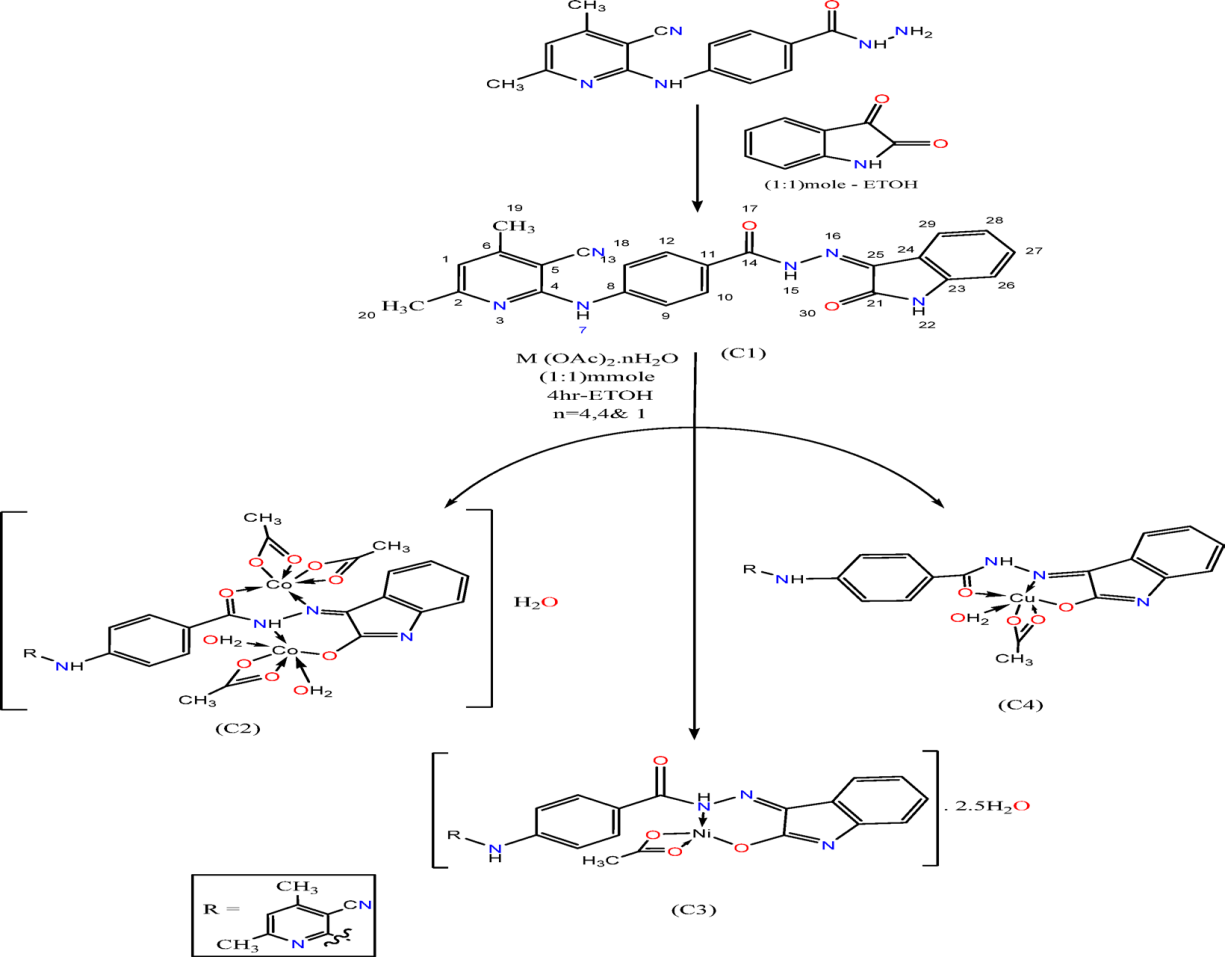
preparation of H_2_BISH and its metal complexes.

The ligand (C1) was synthesized according to the scheme below (Scheme 1) (Structure 1a) by the condensation of 1:1 molar ratio of 4-((3-cyano-4,6-dimethylpyridin-2-yl)amino)benzohydrazide^28^ (0.28 g, 1 mmol) with 0.147 g of isatine (1 mmol) in 15 mL hot ethanolic solution. The reaction mixture was boiled under reflux for 2 h, where a deep yellow precipitate was formed, was filtered off, recrystallized from ethanol and finally dried in a vacuum desiccator over anhydrous CaCl_2_. The purity of the hydrazone, H_2_BISH was checked by TLC, IR and^1^H, ^13^CNMR and mass spectra.

A hot ethanolic solution of the respective metal acetate hydrate salts (1.0 mmol) was added to hot ethanolic solution of (H_2_BISH) (0.410 g, 1.0 mmol). The mixture was heated under reflux for 6 h and the products formed were filtered off, washed with ethanol, diethyl ether and dried in a vacuum desiccator over anhydrous CaCl_2_. The complexes (Structure 1b–e in Scheme 1) are stable in air and insoluble in non-polar solvents but soluble in DMF and DMSO solvents. The analytical data corresponding to the title complexes are recorded in Table 1S (Supplementary Material).

#### H_2_BISH (C1)

C_23_H_18_N_6_O_2_ (410.44) yield: 91%, dark yellow and M.P.: 290 °C Percentage of element. Calculated; C (67.31%); H (4.21%); N (20.48%) Found; C (65.59%); H (4.42%); N (20.04%) FTIR (cm^−1^) 1718 ν(C=O)_isatin_; 1686 ν(C=O); 1559 ν(C=N)_azomethine_; 1609 ν(C=N)_py_; 2218, ν(C≡N).

#### [Co_2_(HBISH)(CH_3_COO)_3_(H_2_O)_2_]·H_2_O (C2)

C_29_H_32_Co_2_N_6_O_11_ (758.48) yield: 95%. Reddish brown; M.P. > 300 °C; Percentage of element. (Calculated%); C (45.92); H (4.25), N (11.08); Co (15.54); Found; C 45.08; H 4.11; N11.32; Co 15.28. FTIR (cm^−1^): 1680 ν(C=O); 1562 ν(C=N)_azomethine_; 1610 ν(C=N)_py_; 2216 ν(C≡N); 1256 ν(C-O)_is_; 1538 new ν(C=N)^*^_isatin_; 508 ν(Co–O); 449 ν(Co–N**)**.

#### [Ni(HBISH)(CH_3_COO)]0.2·5H_2_O (C3)

C_25_H_25_N_6_O_6.5_Ni (572.21) yield: 88%. Yellow; M.P. > 300 °C; Percentage of element; (Calculated%); C (52.48); H (4.40); N (14.69); Ni (10.23). Found; C 52.86; H 4.18; N 14.13; Ni 10.64. FTIR (cm^−1^): 1688 ν(C=O); 1560 ν(C=N); 1613 ν(C=N)_py_; 2217 ν(C≡N); 1255ν(C–O)_is_; 1535 ν(C=N)^*^_isatin_; 505 ν(Ni–O); 451 ν(Ni–N**)**.

#### [Cu(HBISH)(CH_3_COO)(H_2_O)] (C4)

C_25_H_22_CuN_6_O_5_ (550.04) yield: 71%. Reddish Brown; m.p. > 300 °C; Percentage of element. (Calculated%); C (54.59); H (4.03); N (15.28); Cu (11.55); Found; C 55.10; H 4.24; N 16.14 Cu 11.93. FTIR (cm^−1^): 1682 ν(C=O); 1562 ν(C=N); 1609 ν(C=N)_py_; 2212 ν(C≡N); 1256 ν(C–O)_isatin_; 1538 ν(C=N)^*^_isatin;_ 506 ν(Cu–O); 431 ν(Cu–N**)**.

### Biological activity

#### Free radical scavenging power (DPPH)

Antioxidant power of the investigated samples was tested as described by Kitts et al.^29^ utilizing ascorbic acid as standard. The hydrogen atom or electron donation ability of the corresponding compounds was measured from the bleaching of purple colored of the methanolic solution of DPPH. This spectrophotometric assay uses the stable radical diphenylpicrylhydrazyl (DPPH) as a reagent. Different concentrations of the chemical compounds were dissolved in methanol to obtain final concentration ranged from 6.25 to 200 mg/mL to determine IC_50_ (concentration makes 50% inhibition of DPPH color). Fifty microliters of various sample concentrations were added to 5 mL of 0.004% methanolic solution of DPPH. After a 60 min of incubation at dark, the absorbance was read against a blank at 517 nm and the % DPPH^·^ remaining was calculated as:

$$\% {\text{ DPPH}}^{ \cdot } {\text{remaining }} = \, \left[ {{\text{DPPH}}^{ \cdot } } \right]_{{\text{T}}} / \, \left[ {{\text{DPPH}}^{ \cdot } } \right]_{{{\text{T}} = \, 0}} \times \, 100$$

By plotting the % DPPH^·^ remaining values vs mg extract/mL, IC_50_ concentration is obtained which expresses the antioxidants necessary to decrease 50% of the initial DPPH^·^ solution concentration. IC_50_ values indicate inverse relationship with the sample antioxidant activity.

#### Antibacterial inhibition

The compounds under study(C1–C4) were tested for inhibition of growth of Gram + ve, *Bacillus cereus* (*B. cereus*) and Gram − ve, *Escherichia coli* (*E. coli*, ATCC 25922) strains by diffusion method^30^ using agar as nutrient and *Cefotaxime* antibiotic (CTX) (4 μg/mL). The tested compounds were dissolved in DMSO which have no inhibition activity to get concentrations of 200 mg/mL. The nutrient media of the used petri dishes were content agar, beef extract and peptone (25, 3, 5 g, respectively). Paper discs immersed and put in this petri dish contain *B. cereus and Escherichia coli* bacteria species then petri dishes were incubated at 37 °C for 24 h. The inhibition zones were measured in mm.

#### Cytotoxicity assay

MTT((3-(4,5-dimethylthiazol-2-yl)-2,5-diphenyltetrazolium) is used to assess cell viability as a function of redox potential. 100 µL cell suspension (5 × 10^3^ cells) were incubated in 96 well plates in complete media for 24 h at temperature equal 37 °C in 5% CO_2_. Aliquots of 100 µL of drug-containing medium at different concentrations were used to treat the cells. After, 48 h of drug addition, media was neglected, MTT solution that contains 20 µL of 1 mg/mL standard solution was added up to PBS solution (100 µL) and incubated again at (T = 37 °C) for 4 h. Then dissolved the produced formazan crystals in absolute DMSO (100 µL). The λ_max_ absorbance of formazan was recorded at 570 nm with using BMGLABTECH®FLUO star Omega, Germany as multi-well plate reader^31^.

### Molecular modeling

All calculations reported were computed using DMOL^3^ program^31^ in Materials Studio 7.0 package using (DFT semi-core pseudopods (dspp)) computations with the double numerical basis sets plus polarization functional (DNP) which was suggested by Kessi et al. and the revised Perdew–Burke–Ernzerh of functionals (RPBE)^32–34^ to be more accurate when comparison with Gaussian basis sets of the same size.

### Molecular docking

Molecular docking studies were performed using MOE2022 software^35^. To obtain the putative crystallographic structures of breast cancer (PDB ID: 3HB5), *E. coli* (PDB ID: 5I39), and Bacillus cereus (PDB ID: 5ZIY), the Protein Data Bank was used. Numerous docking simulations were applied using the default parameters and the conformations were selected according to the E conformation, S score data and precise fitting with the pertinent amino acids in the binding core. The compounds were permitted to investigate the conformational region within the cavity of the receptor while the focal protein remained rigid.

## Results and discussion

### IR spectra

**Fig. 1:**
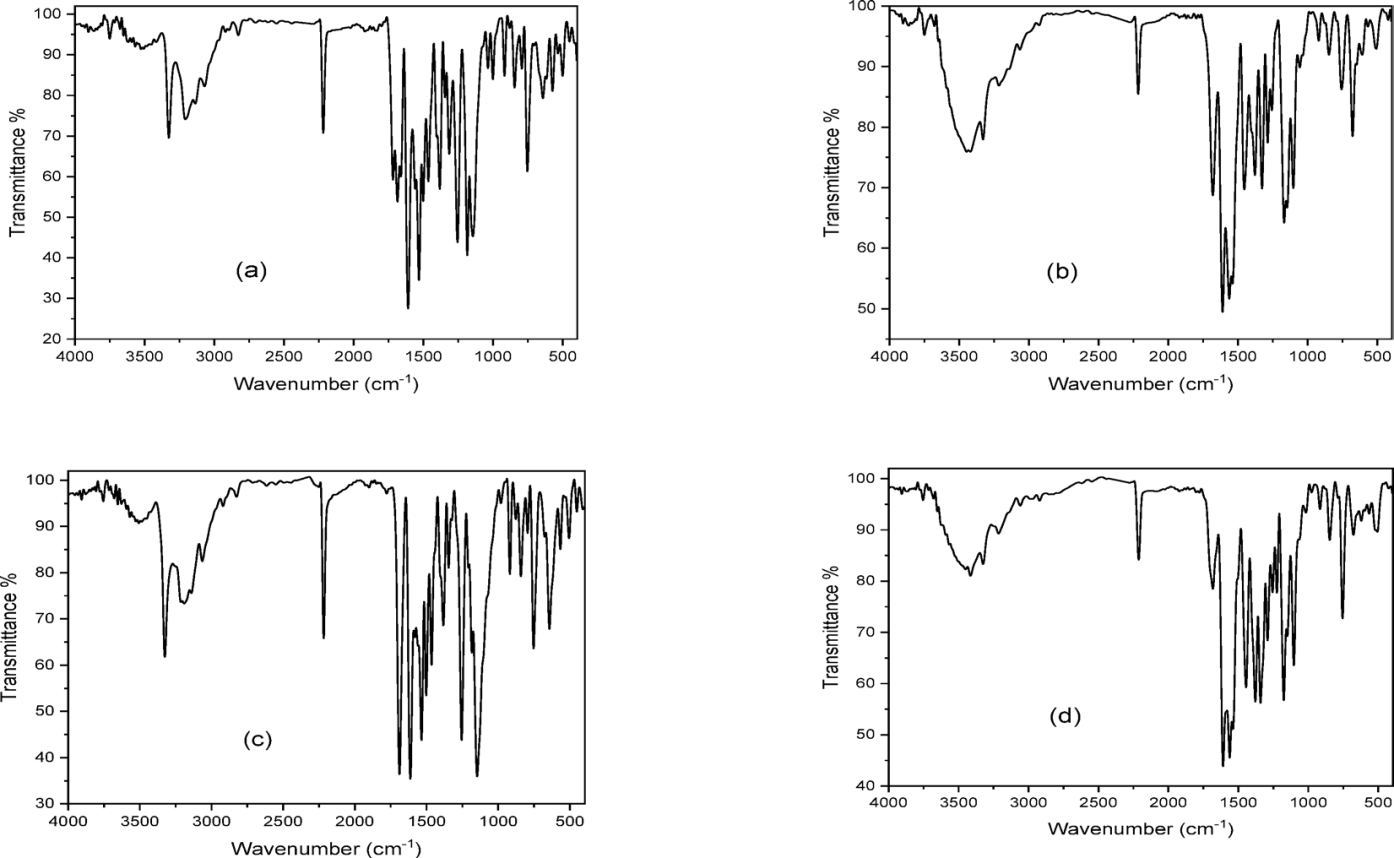
FTIR spectra of (**a**) H_2_BISH (C1), (**b**) [Co_2_(HBISH)(CH_3_COO)_3_(H_2_O)_2_]·H_2_O (C2), (**c**) [Ni(HBISH)(CH_3_COO)]·2.5H_2_O (C3) and (**d**) [Cu(HBISH)(CH_3_COO)(H_2_O)] (C4).

IR spectra of H_2_BISH ligand (C1) (Scheme 1) and its metal complexes are shown in (Table 1 and Fig. 1a–d). The spectrum of free H_2_BISH ligand (Fig. 1a) exhibits strong bands at 1718 and 1686 cm^−1^ are assignable to ν(C=O)_isa_ and (C=O)_hyd_, respectively. The bands at 3327, 3209 and 3137 cm^−1^ are characteristic for ν(NH)_py_, ν(NH)_hyd_ and ν(NH)i_sa_ modes^36^. In the IR spectra of complexes, the bands assigned to ν(C=O)_isa_ and ν(NH)i_sa_ may disappear suggesting enolization of C=O (–C=O → –C–OH) followed by loss of the proton upon complexation whereas ν(NH)_hyd_ may undergo red shift (≈ 18 cm^−1^) or blue shift (≈ 10 cm^−1^) supporting its sharing in coordination to metal ion. The bands observed at 1559 and 1036 cm^−1^ are due to ν(C=N)_azomethine_ and ν(N–N) vibrational modes. The later band suffers shift. The band observed at 1609 cm^−1^, may be due to overlap of (C=N)_py_ and ν(C=C)_ph_ while those appeared at 1001, 642 and 409 cm^−1^ are attributed to the breathing, in-plane and out of plane bending vibrations of the pyridine ring^37^. The sharp band at 2218 cm^−1^ is assigned to the terminal ν(CN) attached to pyridine ring lying at meta position. The weak doublet bands observed at 2890 and 2923 cm^−1^ are due to the symmetric and asymmetric stretching vibrational modes of the two methyl groups in the ortho and para positions to N of pyridine ring.

**Table 1:** Spectral IR data of H_2_BISH ligand (C1) and its complexes (C2–C4).

Compound	ν(C=O)_is_	ν(C=O)_hyd_	ν(NH)_py_	ν(NH)_hy_	ν(NH)_is_	ν(C=N)_azo_	ν(C=N)_py_	ν(N–N)	ν(C–O)_is_	New (C=N)*	ν(M–O)	ν(M–N)
(C1)	1718	1686	3327	3209	3137	1559	1609	1036	–	–	–	–
(C2)	–	1670	3329	3220	–	1576	1610	1048	1258	1538	508	449
(C3)	–	1688	3326	3191	–	1545	1613	1042	1252	1535	505	451
(C4)	–	1672	3325	3219	–	1579	1609	1045	1256	1538	506	468

*ν(C=O)_isa_: of isatin moiety; ν(C=O)_hyd_: of hydraznoic moiety; ν(C=N)_azo_: of azomethine group_;_ ν(C=N)_py_: of pyridine ring.

In the binuclear [Co_2_(HBISH)(CH_3_COO)_3_(H_2_O)_2_]·H_2_O (C2) (Scheme1), the ligand acts as monobasic N_2_O_2_ tetradentate in keto-enol form (Fig. 1b) binding two metal ions. Steric factor sprevent the coordination of the two carbonyls to a single metal ion, therefore; one ion through O atom of (C–O)_isa_ and N atom of (NH)_hyd_ from one side forming a six membered chelate ring surround metal ion and the other metal atom through O atom of (C=O)_hyd_, and N atom of (C=N)_azo_ groups on the other side of hydrazone moiety in a five membered chelate ring as proved by: (1) disappearance of bands due to (C=O)_isa_, (NH)_isa_ with simultaneous of new bands at 1538 and 1258 cm^−1^ assignable to new ν(C=N*) and ν(C–O) suggesting enolization of the carbonyl group and loss of proton on coordination to one metal atom, (2) shift of (NH)_hyd_ (≈ 11 cm^−1^) to higher wavenumber; (3) additional evidence for suggested mode of coordination is the presence of a bands at 449 and 508 cm^−1^ due to ν(Co–N) and ν(Co–N) vibration for the divalent metal centers^37^.

H_2_BISH ligand acts as monobasic NO bidentate in [Ni(H_2_BISH)(CH_3_COO)]0.2·5H_2_O complex (C3) (Fig. 1c) through the N atom of (C=N)_azo_ and the oxygen atom of (C–O)_isa_ groups formed as result of enolization followed by deprotonation on coordination. This suggestion is supported by: (1) The bands refer to the ν(C=O)_is_ and ν(NH)_isa_ disappeared with the appearance of new bands at (1255 cm^−1^) and (1535 cm^−1^) assigned to ν(C–O) and new ν(C=N*)_isa_ mode; (2) The band assigned to ν(C=N)_azo_ shifted to higher wavenumber (1582 cm^−1^) confirms suggested coordination mode^37^ which is further supported by the increase in frequency of the hydrazinic N–N bond as a consequence of the reduction between the lone pairs of electrons and (3) New bands at 505 and 451 cm^−1^ attributed to ν(M–O) and ν(M–N) modes.

On the other hand, the hydrazone, H_2_BISH behaves as monobasic ONO tridentate in [Cu(HBISH)(CH_3_COO)(H_2_O)] (C4) (Scheme1) complex (Fig. 1d) via enolized (C–O)_isa_, (C=N)_azo_ and (C=O)_hyd_ groups^37^ as revealed by: (1) Red shift of bands characteristic for ν(C=N)_azo_ (≈ 20 cm^−1^) and ν(N–N) (≈ 9 cm^−1^) is evidence of coordination through azomethine nitrogen, (2) Disappearance of ν(C=O)_is_ and ν(NH)_isa,_ instead new bands appeared at 1256 cm^−1^ and 1538 cm^−1^ assignable to (C–O)_isa_ and new ν(C=N*)_isa_ (3) New bands at 506 cm^−1^ and 468 cm^−1^ due to ν(M–O) and ν(M–N) modes confirming postulated coordination mode.

The acetate groups in the titled complexes (C2), (C3) and (C4) coordinated as bidentate ligand that confirmed by the bands appeared at (1454, 1327); (1445, 1341) and (1464, 1346) cm^−1^. This bands characterize ν_asy_(OCO) and ν_sy_(OCO) with wavenumber difference values (Δυ = 127, 104 and 118 cm^−1^), respectively. The differences calculated match with the reported values in^38^.

The bands observed in the regions ≈ 3400–3456, 862–850, and at that 577 cm^−1^ observed as broad ones in the spectra of the investigated complexes are referred to ν(OH), ∆(H_2_O), p_r_(H_2_O) and P_w_ (H_2_O) vibrations for the coordinated water. Also, the broad band centered at 3500 cm^−1^ in the spectra of the studied complexes may be due to hydrated water. To verify between the coordinated and hydrated water, TGA measurement was carried out^38^.

### NMR spectral study

**Fig. 2:**
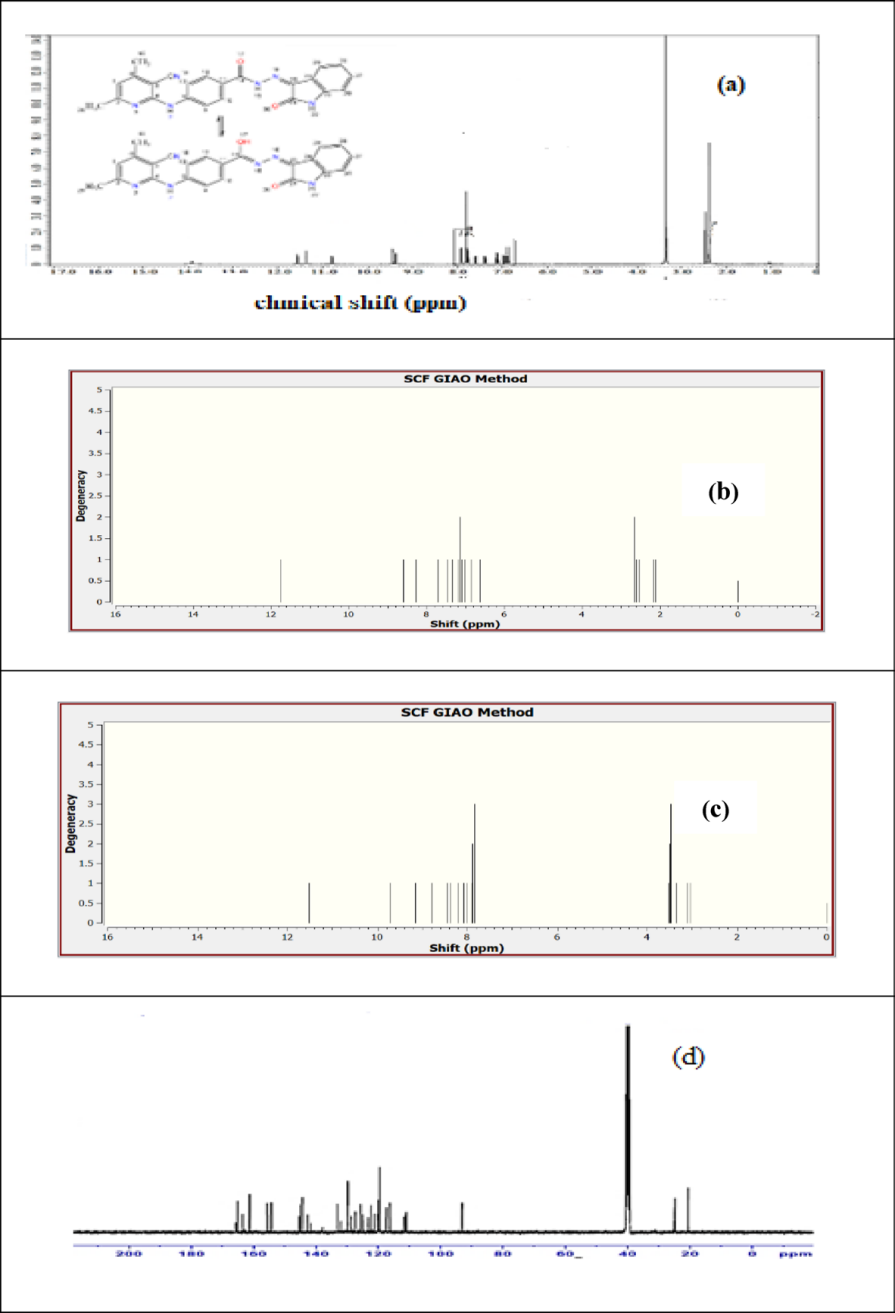
Experimental ^1^H-NMR (**a**), Theoretical ^1^H-NMR spectra for keto form (**b**), Theoretical ^1^H-NMR spectrum for enol form (**c**) and ^13^CNMR spectrum of H_2_BISH (**d**).

^1^HNMR spectrum of the ligand, H_2_BISH (C1) (Fig. 2a and Table 2) showed the appearance of peaks corresponding to four protons of isatin moiety twice at δ_H_ (6.92, 7.10, 7.41 and 7.61 ppm) and (6.97, 7.13, 7.39 and 7.94 ppm) for H-26, H-28, H-27, and H-29, respectively which indicated the presence of two isomers forms (Keto-enol). Two AA′BB′ systems (protons of benzene rings) (Fig. 2a) were found in two different positions one at δ_H_ 7.83 ppm as singlet for four protons (4H on C-9, C-10, C-12, C-13) overlapped on each other and the other one at δ_H_ 7.79 ppm (d, J = 8.8 Hz, 2H-C-10, C-12) and 7.94 ppm (d, J = 8.8 Hz, 2H-C-9, C-13). Also, two singlet signals for the same protons (H-C-1) in pyridine ring appeared at 6.89 and 6.90 ppm. In addition, the methyl groups attached to the pyridine ring appeared twice at 2.41 and 2.42 ppm^39^. The above-mentioned data indicated the presence of two isomers ketone and enol forms at the carbonyl of hydrazine (C-14). Ketone form contains three NH groups recorded at δ_H_ 9.39, 10.84, and 11.58 ppm for NH-7, NH-22, and NH-15, respectively. Enol form structure contains two NH groups and one OH resulting from enolization of hydrazonoic C=O group (C-14) group recorded at δ_H_ 9.45, 11.38, and 13.94 ppm for NH-7, NH-22, and OH-15, respectively. The ratio between enol and ketone form was 1.25:1.00, respectively. ^13^C-NMR spectrum confirmed the presence of the two isomers showed in Fig. 2b. The carbon spectral signal for the hydrazine carbonyl of the first isomer was located at 163.6 ppm, the absorption signal of the second isomer C–OH was found at 155.4 ppm. The other spectral signals of isatin carbonyl, C(2), C(4), C(5) and C(6) at (165.3, 161.3, 154.2, 93.1 and 155.6 ppm) appear as two signals for two carbon atoms assignable to the two isomers overlapping. These experimental results supported by the DFT theoretically calculated values are as shown (Fig. 2b, c).

**Table 2:** ^1^H NMR data of keto-enol forms of H_2_BISH.

Proton	Keto form		Enol form	
	Chemical shift (ppm)	J coupling (Hz)	Chemical shift (ppm)	J coupling (Hz)
3H-C19, 3H-C20	(s) 2.41, 2.40	–	(s) 2.41, 2.40	–
	**2.18, 2.67**		**3.11, 3.51**	
H-C1	(s) 6.89	–	(s) 6.90	–
	**6.63**		**7.84**	
H-C9 & H-C13	(s) 7.83	–	(d) 7.94	8.8
	**7.26**		**8.30**	
H-C10 & H-C12	(s) 7.83	–	(d) 7.79	8.8
	**7.58**		**8.60**	
H-C26	(d) 6.91	8	(d) 6.97	7.5
	**6.87**		**7.88**	
H-C27	(m) 7.41	–	(m) 7.39	–
	**7.10**		**7.99**	
H-C28	(d) 7.10	7.5	(d) 7.13	7.4
	**7.01**		**7.90**	
H-C29	(d)7.61	8.5	(d)7.94	8
	**7.16**		**8.09**	

*Bold: theoretical chemical shift obtained by TMS HF/6-31G(d) GIAO.

### Electronic spectra

**Fig. 3:**
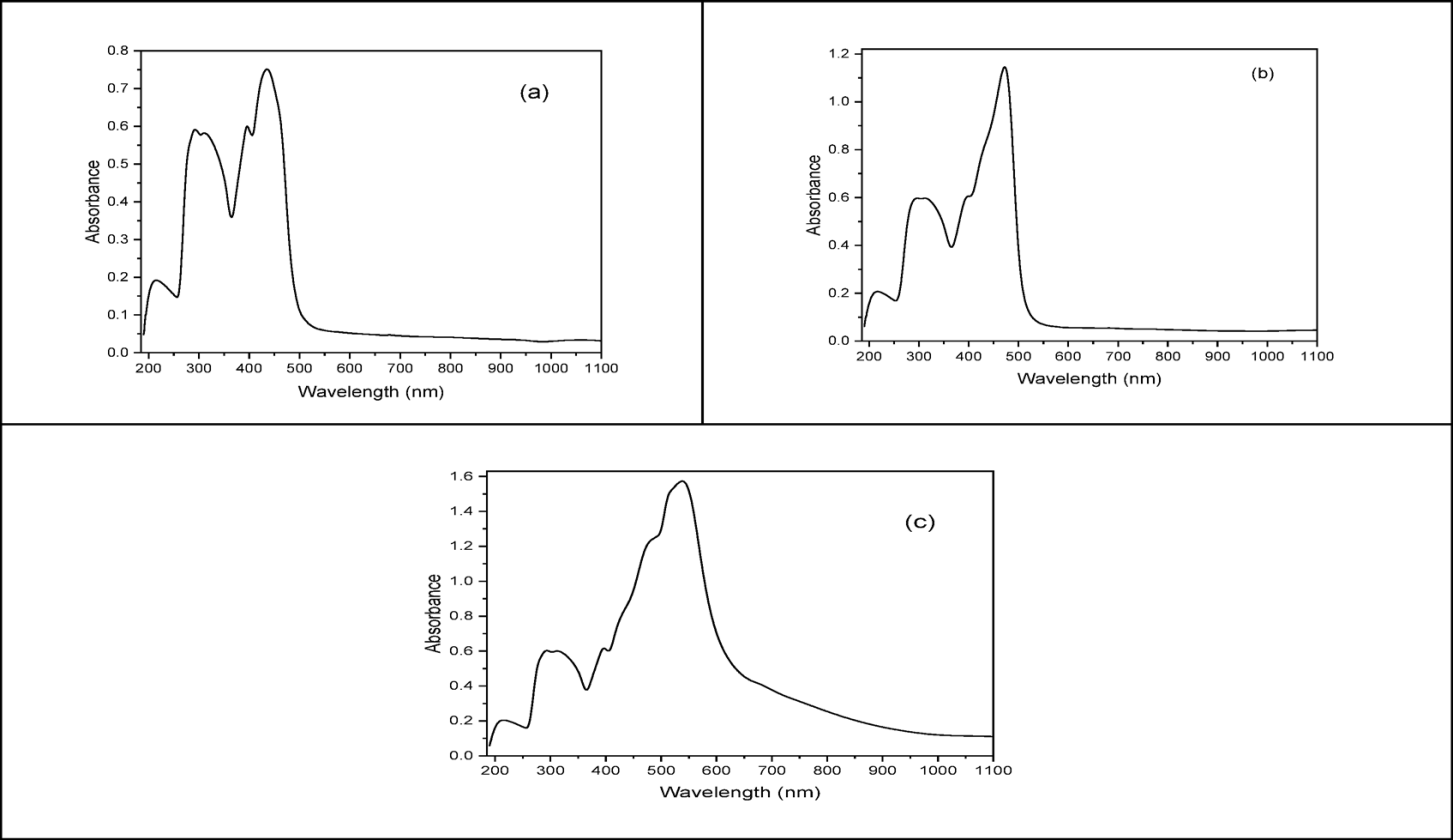
Ultraviolet-vis. spectra of (**a**) H_2_BISH (**b**) [Ni(H_2_BISH)(CH_3_COO)]·2.5H_2_O (**c**) [Cu(HBISH)(CH_3_COO)(H_2_O)].

The electronic spectra of H_2_BISH ligand (C1) and its complexes (C2–C4) were displayed in DMF as solvent. During measurement, we examined the effect of solvent on the absorption spectra of metal complexes by carrying the measurement using Nujol or other solvents such as DMSO, no change was observed. These spectra are illustrated in Fig. 3 and the data obtained are represented in Table 3. The ligand spectrum displayed distinct bands at (215, 292 nm) (46,511, 34,246 cm^−1^) and (311, 396 nm) (32,154, 25,252 cm^−1^) are attributable to (π → π*) and (n → π*) transitions, referring to the azomethine group and the aromatic rings, respectively. The intraligand charge transfer band appeared at λ max = 436 nm (22,935 cm^−1^). These transitions showed red or blue frequencies shifts because of the coordination of the H_2_BISH ligand with the divalent metal ions^40^.

**Table 3:** Electronic spectral data, magnetic moments and optical band gap energy values of H_2_BISH (C1) and its metal complexes (C2–C4).

Compound	Band position (cm^−1^)	Assignment	Ligand field parameters			μ_eff_ (B.M)	λ_cutoff_	Optical band gap (eV)		
			B	Β	∆_q_			E_opt_***	Direct	Indirect
(C1)	46,511, 34,246 32,154, 25,252	π → π* n → π*	–	–	–	–	–		–	–
(C2)	**46,728** **,** **34,129** **31,746** **,** **25,188** 20,202 16,447	**π → π** * **n → π** * ^4^T_1_g → ^4^T_1g_(P) ^4^T_2_g → ^4^A_2g_	697.5	0.72	788.1	4.96**	413	3.002	3.221	3.041
(C3)	**46,082** **,** **33,898** **31,948** **,** **25,125** 14,727	**π → π** * **n → π** * ^3^T_1_(F) → ^3^T_1_(P)	–	–	–	2.80	435	2.851	3.164	2.850
(C4)	**46,511** **,** **34,129** **31,847** **,** **25,188** 20,876 12,285	**π → π** * **n → π** * 2B_1g_ → 2E_g_ 2B_1g_ → 2A_1g_	–	–	–	1.85	414	2.995	3.226	2.912

**Value of μ_eff._ per one atom.

***Values calculated from λ_cutoff_.

The electronic spectrum of [Co_2_(HBISH)(CH_3_COO)_3_(H_2_O)_2_]·H_2_O complex (C2) exhibits bands at (675 nm) (14,814 cm^−1^) and (608 nm) (16,447 cm^−1^) refers to ^4^T_2_g → ^4^A_2g_ (ν_2_) and ^4^T_1_g → ^4^T_1g_(P) (ν_3_) in an octahedral environment. The calculated ligand field parameters Dq, B and β values were 788.1 cm^−1^, 697.5 cm^−1^, and 0.72, respectively. The magnetic moment value (4.96 B.M.) per one metal ion which is higher than those reported for high spin octanedral Co(II) complexes that may be referred to orbital contribution^40^.

The [Ni(H_2_BISH)(CH_3_COO)]0.2·5H_2_O (C3) electronic spectrum exhibits a significant band at (679 nm) (14,727 cm^−1^) appropriate to ^3^T_1_(F) → ^3^T_1_(P) transition in tetrahedral geometry of the nickel atom. The calculated magnetic moment value is (2.80 B.M.)^41^.

The spectrum of copper (II) complex (C4) indicates a red shift in the bands of the ligand after coordination in addition to new bands appeared at 12,285 (814 nm) and 20,876 cm^−1^ (479 nm) assignable to ^2^B_1g_ → ^2^E_g_ (ν_2_) and ^2^B_1g_ → ^2^A_1g_ (ν_3_) transitions in the octahedral geometry^41^. The magnetic moment equals 1.85 B.M.

### Optical band gap

**Fig. 4:**
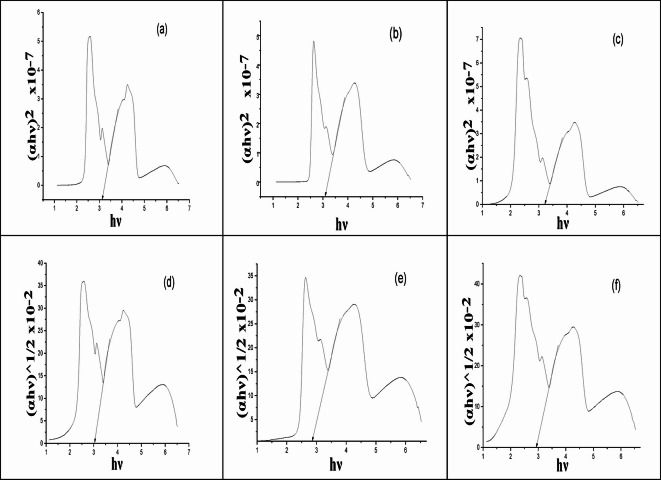
Optical band gap energies of direct transitions (**a**–**c**) and in-direct transitions (**d**–**f**) for i) [Co_2_(HBISH)(CH_3_COO)_3_(H_2_O)_2_]·H_2_O (C2), ii) [Ni(HBISH)(CH_3_COO)]·2.5H_2_O (C3) and iii) [Cu(HBISH)(CH_3_COO)(H_2_O)] (C4) complexes.

It is known that metal complexes played a significant role in solar cell as harvesting materials due to their abilities to absorb radiation in both UV and visible regions^42^. To clarify the conductivity behavior of the metal complexes under study, the optical band gap (E_g_) have been calculated from their absorption spectra (Fig. 3b–d) by applying Tauc’s equation,

1 $$\left( {\alpha h\nu } \right) = A\left( {h\nu - E_{g} } \right)^{m}$$

where A is an energy-independent constant, h is Planck’s constant (4.14 × 10^−15^ eV/s m is a constant, determines the transition type, as it equals (0.5) for direct allowed transition and (2) for indirect allowed transition, ⍺ is the absorbance coefficient and was estimated from the formula:

2 $$\alpha = 1/dln A$$

where d is the thickness of the filter paper, A is the absorbance value. On plotting (⍺hν)^2^ versus (hν) (Fig. 4a–c), the E_g_ value for the allowed direct transition can be obtained from the intersection of the straight line of the curve with x-axis which represents the values of (hν). The data in Table 3 indicated that the straight-line plots were obtained at *m* = 2 for metal complexes suggesting direct allowed transition mechanism. The optical band gap values of the complexes exhibited lower values, 3.16, 3.221 and 3.23 eV which may attributed to decrease in HOMO–LUMO energy gap on complex formation in which ligand and metal orbitals overlapped. In addition, the E_g_ obtained values were comparable to those of efficient photovoltaic materials and suggested that the ligand and its complexes have semi-conductive nature^43^.

### Mass spectra

**Fig. 5:**
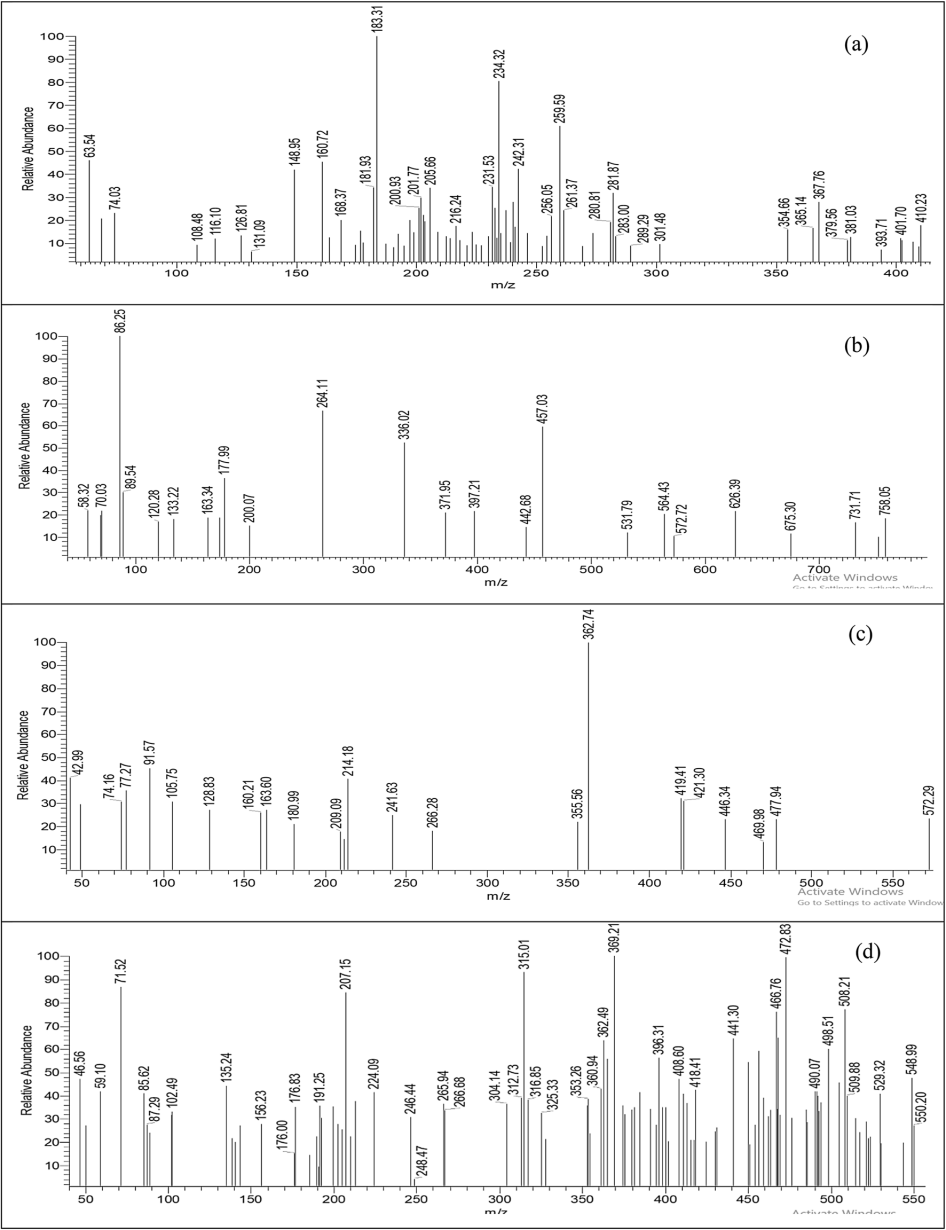
Mass spectra of (**a**) H_2_BISH (C1); (**b**) [Co_2_(HBISH)(CH_3_COO)_3_(H_2_O)_2_].H_2_O (C2), (**c**) [Ni(HBISH)(CH_3_COO)].2.5H_2_O (C3) and(**d**) [Cu(HBISH)(CH_3_COO)(H_2_O)] (C4) complex.

**Scheme 2:**
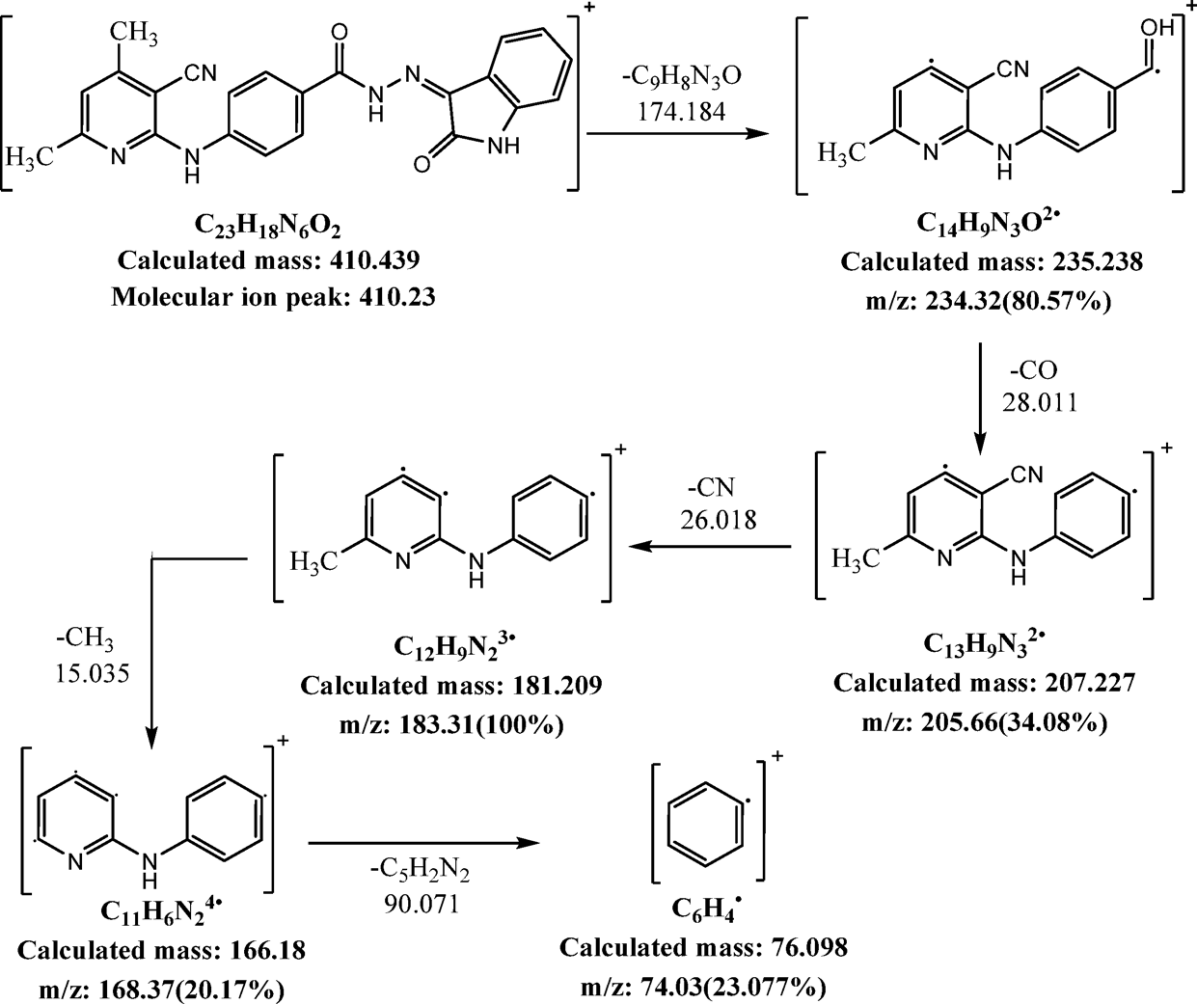
The fragmentation patterns of: (**a**) H_2_BISH ligand(C1); (**b**) Co(II) complex (C2); (**c**) Ni(II) complex (C3) and **(d)** Cu(II) complex (C4).

The mass spectra of H_2_BISH ligand (C1) and its metal complexes (C2–C4) are illustrated in Fig. 5a–d and Scheme 2a–d show its fragmentation patterns. The H_2_BISH ligand spectrum (Fig. 5a) showed the molecular ion peak (M^+^) at m/z = 410.23 (*Rel.int*; 17.87%) matched with the molecular weight (410.439). The maximum peak is corresponding to C_12_H_9_N_2_ fragment at m/z = 183.31 (*Rel.int*; 100.00%). The peak at m/z = 234.32 (*Rel.int*; 80.57%) represents the C_14_H_9_N_3_O fragment. The peak at m/z = 205.66 (*Rel.int*; 34.08%) corresponds to C_13_H_9_N_3_ fragment. The peak at m/z = 168.37 (*Rel.int*; 20.17%) belongs to C_11_H_6_N_2_ fragment. Finally, the peak at m/z = 74.03 (*Rel.int*; 23.077%) is related to C_6_H_4_ fragment. The mass spectra of the complexes (Fig. 5b–d) exhibited the molecular ion peaks (M^+^) at m/z = (758.05, 572.29 and 550.20) with *Rel.int;* 18.33%, 23.19% and 27.07%, respectively which are consistent with the calculated molecular weights of the investigated complexes (758.48, 572.21 and 550.03), respectively. The peaks observed at *Rel.int*; 100.00%) are corresponding to m/z = 86.25, 362.74 and 369.21 that assigned to the fragments CoN_2_, C_17_H_11_N_3_NiO_3_ and C_21_H_14_N_5_O_23_, in the spectra of Co(II) (C2), Ni(II) (C3) and Cu(II) (C4) complexes, respectively. The fragmentation pattern of Co(II) complex (C2) indicates various peaks with different m/z values as: 564.43 (20.38%), 457.03 (59.46%), 336.02 (52.44%) and 177.99 (36.24%) which corresponding to C_25_H_24_CoN_6_O_6_, C_21_H_13_CoN_6_O_3_, C_15_H_10_CoN_4_O_2_ and C_7_H_5_CoN_2_ fragments, respectively. The fragmentation pattern of Ni(II) complex (C3) exhibits the following fragments: C_23_H_16_N_5_NiO_3_ (m/z = 469.98 (13.27%)), C_9_H_5_N_3_Ni (m/z = 214.18 (40.44%)) and CHN_2_ (m/z = 42.99 (40.95%)). On the other hand, the mass spectrum of Cu complex (C4) peaks at (m/z = 490.07 (41.65%)), (m/z = 472.83 (99.44%)), (m/z = 207.15 (84.55%)) and (m/z = 88.82 (23.84%)). These peaks are attributed to C_23_H_19_CuN_6_O_3_, C_23_H_17_CuN_6_O_2_, C_12_H_5_N_3_O and C_5_HN_2_ fragments, respectively.

### ESR spectral study

In case of octahedral, square pyramidal, or square planar geometries of Cu (II) complexes, the ground state is the *d*_*x*2*–y*2_ giving ^2^B_1g_ as the ground term. The ESR spectroscopy can distinguish the ground states based on the principle values of the g-tensor, where g_||_> g_⊥_ > 2.0023 when ^2^B_1g_ is the ground state, while in the case of ^2^A_1g_ ground state the g_⊥_ > g_||_> 2.0023^42^.

**Fig. 6:**
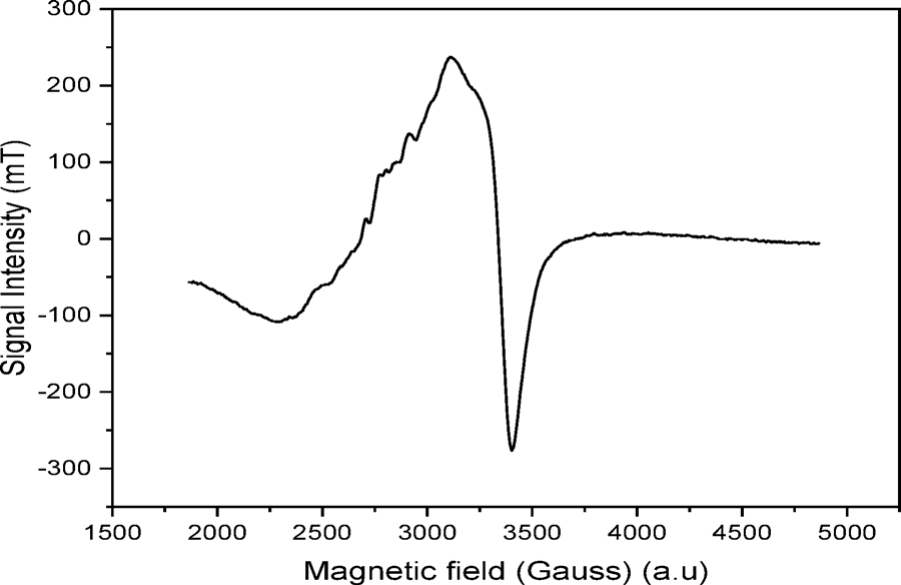
ESR spectrum of [Cu(HBISH)(CH_3_COO)(H_2_O)] (C4) complex.

The solid-state ESR spectrum of [Cu(HBISH)(CH_3_COO)(H_2_O)] (C4) complex at room temperature using the DPPH as calibrating substance (Fig. 6) that is a single line concentrated at g_iso_ = 2.142 accompanying with characterized hyperfine structure, indicating that the hydrazone (ONO) chelated system observes to be coplanar with two chelating rings around the metal ion (Scheme 1) which agree with the planar octahedral geometry like those found in literature about mononuclear copper^42^ exhibiting g-tensor values at g_||_= 2.290 and g_⊥_ = 2.068, respectively. The obtained g-values were g_||_> g_⊥_ > 2.0023 which indicates that the Cu(II) has a *d*_*x*2*–y*2_ ground-state ground state characteristic for the octahedral or square planar geometry^43^. The observed values of g_||_ is less than 2.3 suggesting a significant covalent character of metal–ligand bonds. The exchange interaction between Cu(II) centers in a solid state may be expressed by the axial symmetry parameter, G, where G = (g_||_ − 2)/(g_⊥_ − 2). If G > 4, the exchange interaction is negligible, whereas when G < 4, a considerable exchange interaction is indicated. In the present study, G value (4.26) is more than 4, suggesting that the exchange interaction occur between Cu(II) cores can be neglected. This is supported by the normal value of magnetic moment of the Cu(II) complex (1.85 B.M.). The in-plane (σ-bonding) and β^2^ (π-bonding) molecular orbital coefficient (α^2^) was found to be 0.78 and 0.77, respectively referring that in-plane (σ-bonding) is more ionic and in a great matching with the literature^43^. The observed g_ll_ and A_ll_ (141 × 10^−4^), values come to an agreement with octahedral Cu(II) complex in the environment of nitrogen donor ligands notified elsewhere^43^. To determine the distortion degree of the Cu(II) complex, the f-factor g_||_/A_||_ was found to be 162 revealing a large strong tetrahedral distortion^43^.

In addition, the orbital reduction factor, K, was used as a measure of covalency, where for ionic environment K = 1, and for covalent K < 1. The orbital reduction factor is calculated using the following simplified expressions^44^ where K_ll_ and K_⊥_ are the parallel and perpendicular components of orbital reduction factor, respectively, λ_0_ is the spin–orbit coupling constant (828 cm^−1^) and the d–d transition is obtained from the electronic spectrum. For pure σ-bonding, K_ll_ ≈ K_⊥_ ≈ 0.77 and for in-plane π-bonding K_ll_ < K_⊥_, while for c K_ll_ > K_⊥_^44^.

$$\begin{aligned} & K_{\parallel }^{2} = \frac{{\left( {g_{\parallel } - 2.0023} \right)}}{{8 \times \lambda_{0} }} \times {\text{d}} - {\text{d}}\;transition \\ & K_{ \bot }^{2} = \frac{{\left( {g_{ \bot } - 2.0023} \right)}}{{8 \times \lambda_{0} }} \times {\text{d}} - {\text{d}}\;transition \\ &K^{2} = \frac{{\left( {{\text{K}}_{\parallel }^{2} - 2K_{ \bot }^{2} } \right)}}{3} \\ \end{aligned}$$

The obtained data indicated the acetate copper complex has K_ll_ = 0.53 and K_⊥_ = 0.49, which is indicated that both has K_ll_ > K_⊥_ and so they in-plane π-bonding is slightly more ionic and vice versa for out of π-bonding^44^.

### X-ray powder diffraction

**Fig. 7:**
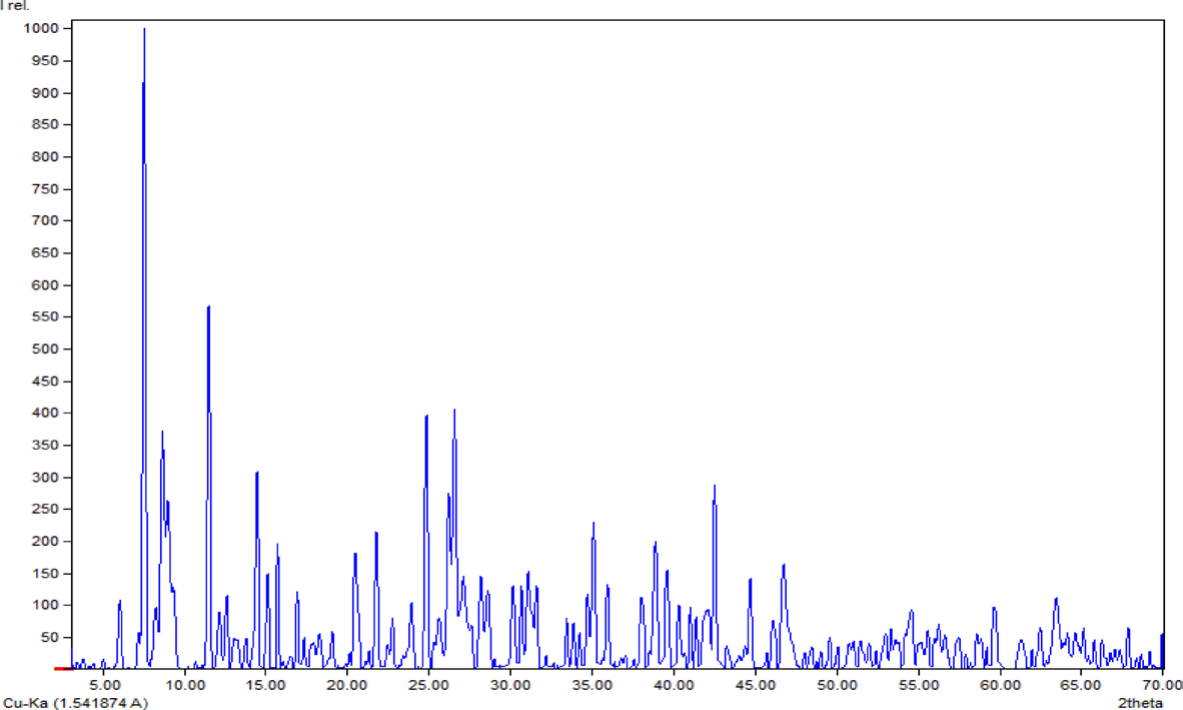
X-ray powder diffraction spectrum of [Co_2_(HBISH)(CH_3_COO)_3_(H_2_O)_2_]·H_2_O complex (C2).

The XRD pattern of [Co_2_(HBISH)(CH_3_COO)_3_(H_2_O)_2_]·H_2_O complex is illustrated in Fig. 7 and the data obtained is reported in Table 4. The diffraction pattern was perfectly identical to the orthorhombic C_30_ Co_3_ N_22_ O_5_ geometry with lattice constants a = 6.5688 Å, b = 31.7110 Å and c = 18.1864 Å and C m c m space group^45^. These results are consistent with the Joint Committee for Powder Diffraction Studies (JCPDS) Entries No. 96-704-5959. Finally, several sharp peaks observed at (7.54°, 12.11°, 21.79°, 31.60°, 34.25° and 46.03°) the spectrum of aforementioned complex.

**Table 4:** Powder XRD data: FWHM, area, intensity, d-spacing, crystallite (grain) size (D), dislocation density, of [Co_2_(HBISH)(CH_3_COO)_3_(H_2_O)_2_]·H_2_O complex (C2).

No.	2theta (°)	hkl	FWHM	d (Å)	Grain (Cystallite) size D (nm)	Lattice strain (ε) × 10^−3^	Dislocation density δ (nm)^−1^ × 10^−4^	Average inner crystal separation (S)
1	7.54	0,2,1	0.18	11.72	45.34	12.1	4.86	14.64
2	12.11	0,4,1	0.18	7.31	46.36	7.40	4.65	9.12
3	14.48	1,1,1	0.18	6.12	46.29	6.21	4.67	7.64
4	15.72	0,2,3	0.13	5.64	63.31	4.18	2.49	7.04
5	21.79	1,3,3	0.18	4.08	46.02	4.16	4.72	5.09
6	28.19	2,2,1	1.64	3.17	5.22	28.5	367.00	3.95
7	31.04	1,7,4	0.38	2.88	22.46	6.02	19.8	3.60
8	31.60	0,4,6	0.17	2.83	49.73	2.67	4.04	3.53
9	33.44	0,8,5	0.11	2.68	77.70	1.62	1.66	3.35
10	34.25	2,24	0.11	2.62	81.29	1.51	1.51	3.27
11	34.69	0,1,0,4	0.17	2.59	51.56	2.36	3.76	3.23
12	35.12	0,2,7	0.20	2.56	44.38	2.70	5.08	3.19
13	46.03	0,8,8	0.26	1.97	34.67	2.67	8.32	2.46
14	46.80	1,7,8	0.38	1.94	23.90	3.81	17.5	2.42
Average	–	–	0.31	4.15	45.59	6.14	32.1	5.18

The morphological parameters as crystallite (grain) size (D), crystal strain (ε) and dislocation density were calculated. Scherer’s formula was utilized to determine the composite’s crystallite grain size (D); D = 0.9λ/(FWHM. Cosθ) where λ = 1.54 Å and (FWHM) refer to the full width of half maximum intensity. The crystalline sizes (D) of compound (3) ranged from 5.22 to 81.29 Å with average (Dav) = 45.59 Å. The lattice strain (ε) is computed by using equation: ε = *FWHM*/4*tan*θ. The values range of (ε) was found to be 1.51 × 10^−3^–2.85 × 10^−2^. The dislocation density value and other XRD characterization parameters are recorded in Table 4.

### Thermal analysis

#### Thermogravimetric studies

The TGA curves of H_2_BISH (C1) and its complexes (C2–C4) were depicted in Fig. 1S (supplementary material). The thermal degradation stages, decomposed fragments and their percentages of all compounds are recorded in Table 5. The ligand (C1) undergoes decomposition through two steps. The first step begins at 327 °C and ends at − 393 °C corresponding to loss of C_8_H_9_N_5_O fragment followed by loss of C_12_H_9_NO in the second one in temperature range (497–658 °C) after which 3C remains as the residual part. For chelates (C2–C4), the first degradation stage in metal complexes generally occurred before 203 °C presumably due to loss of lattice or coordinated water molecules. After the dehydration, the chelate fragments started the decomposition process and at end the remaining residue is either C atoms, metal oxide or both^46^. The [Ni(H_2_BISH)(CH_3_COO)]0.2·5H_2_O (3) thermogram indicates three decomposition steps ending with metal oxide (NiO) as residue at T > 600 °C. The first decomposition step showed the remove of 2.5 mol of hydrated water at 60–126 °C fragments with 7.60% weight loss (Calcd. 7.87%). The second step (309–382 °C) with weight loss (Found % 40.37; CalCd % 40.28) which could be due to the elimination of C_2_H_3_O_2_ + C_9_H_5_N_3_O fragments. The third step at 450–599 °C is attributed to removal of elated to the removal of the fragment (C_14_H_12_N_3_) with weight loss (Found % 39.11; CalCd % 38.84). The residual part is NiO. As a general feature, the residual part is MO or MO in addition to carbon revealing the stability of chelates^47^.

**Table 5:** The temperature range and weight loss of decomposition steps for H_2_BISH (C1) and its metal complexes (C2–C4).

Compounds	Decomposition steps	Temperature range (°C)	Removed species	Wt loss (%) Found% (calculated%)
(C1)	1st	327–393	–C_8_H_9_N_5_O	46.63 (46.58)
	2nd	497–658	–C_12_H_9_NO	44.67 (44.64)
	Residue	659–800	3C	8.70 (8.78)
(C2)	1st	23–68	–H_2_O	2.46 (2.375)
	2nd	69–203	–H_2_O	2.36 (2.375)
	3rd	204–320	–H_2_O + 2CH_3_COOH	17.74 (18.21)
	4th	321–378	–C_5_H_9_N_2_O_2_	16.98 (17.03)
	5th	379–482	–C_20_H_9_N_4_	40.09 (40.25)
	Residue	483–800	2CoO	20.37 (19.76)
(C3)	1st	60–129	–2.5H_2_O	7.60 (7.87)
	2nd	309–382	–C_2_H_3_O_2_ + C_9_H_5_N_3_O	40.37 (40.28)
	3rd	450–599	–C_14_H_12_N_3_	39.11 (38.84)
	Residue	> 600	–NiO	12.92 (13.05)
(C4)	1st	194–257	–H_2_O	3.51 (3.28)
	2nd	299–385	–CH_3_COOH + C_9_H_8_N_5_O	48.07 (47.67)
	3rd	387–448	–C_12_H_8_N	30.24 (30.22)
	Residue	449–800	CuO + 2C	18.18 (18.82)

#### Kinetic studies

The methods used to determine the thermodynamic parameters of each step of the thermal degradation are the Coats–Redfern and Horowitz–Metzger^48,49^. The first order for the number of complexes decomposition has been expected. The thermodynamic parameters (E_a_, ∆H^*^, A, ∆S^*^ and ∆G^*^) for the different thermal degradation steps are calculated by the following equation:

$$\Delta {\text{H}}^{*} = {\text{ E}}_{{\text{a}}} - {\text{RT}},\;\Delta {\text{S}}^{*} = { 2}.{3}0{3}\left( {{\text{log Ah}}/{\text{K}}_{{\text{B}}} {\text{T}}_{{\text{S}}} } \right){\text{ R}}\;{\text{and}}\;\Delta {\text{G}}^{*} = \, \Delta {\text{H}}^{*} - {\text{T}}\Delta {\text{S}}^{*} .$$

**Fig. 8:**
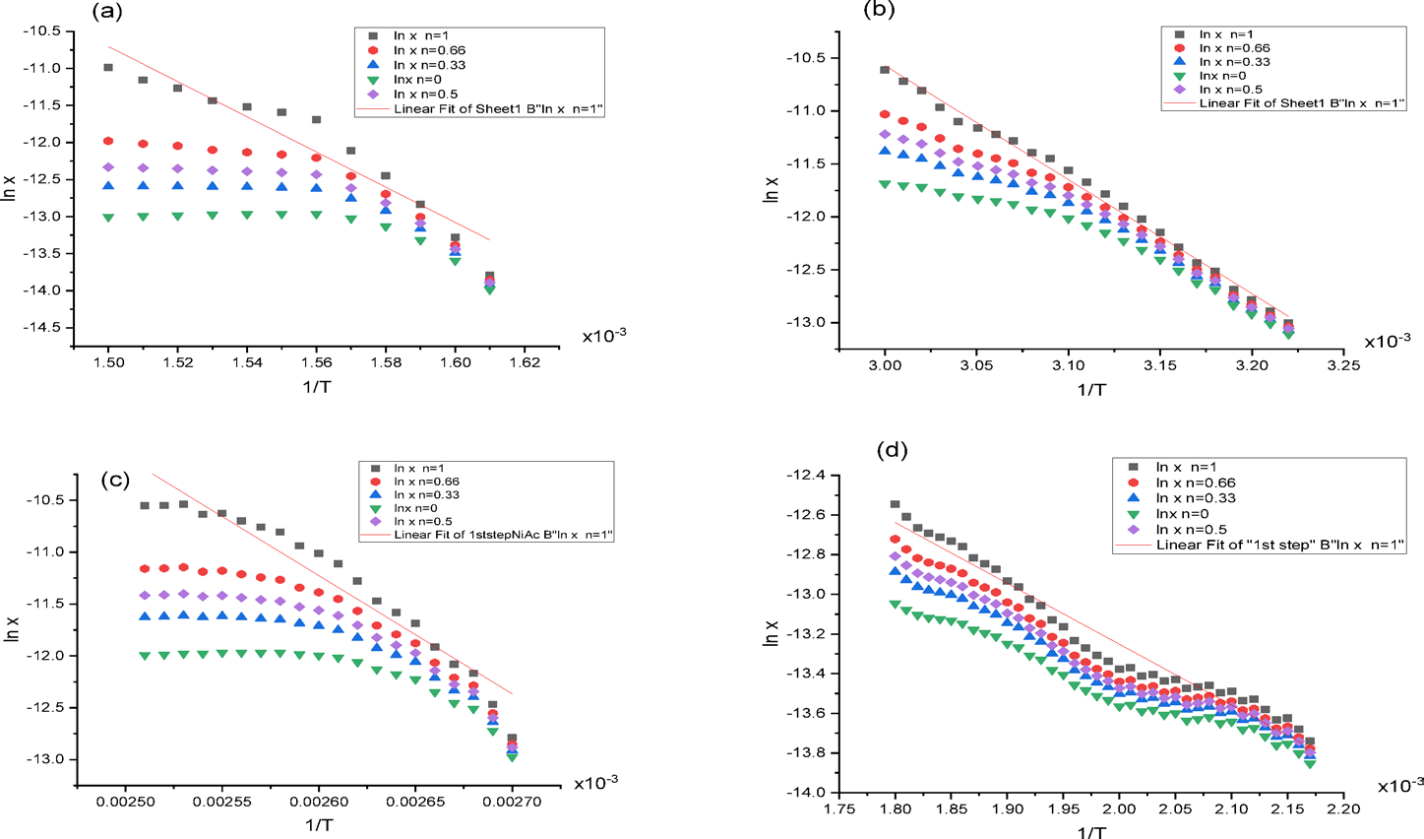
Coats Redfern plots of the first degradation step for (**a**) H_2_BISH, (**b**) [Co_2_(HBISH)(CH_3_COO)_3_(H_2_O)_2_]·H_2_O, (**c**) [Ni(H_2_BISH)(CH_3_COO)]·2.5H_2_O and (**d**) [Cu(HBISH)(CH_3_COO)(H_2_O)].

Data are recorded in Table 6 and reported graphically in (Figs. 8 and 2S). A glance to the table, we found that the activation energy high values reveal the high stability of the compounds indicating a covalent bond character. The decomposition steps of all compounds are not spontaneous due to large positive ΔG^*^ values. Also, the negative ΔS^*^ values are an indication of the low internal energy while the positive magnitudes of ∆H^*^ reveal the endothermic nature of thermal decomposition process.

**Table 6:** Kinetic parameters evaluated by Coats-redfern and Horowitz-Metzger equations of (a) H2BISH (C1) and its metal complexes (C2-C4).

Compound	Peak	Mid temp (K)	Ea (KJ/mol)	A (S^−1^)	∆H* (kJ/mol)	∆S* (kJ/mol K)	∆G* (kJ/mol)
(C1)	1st	643.89	181.17	1.21E+15	175.81	0.0374	151.73
			207.13*	2.48E+15*	201.77*	0.0434*	173.84*
	2nd	883.96	179.46	1.76E+13	172.11	− 0.0004	172.46
			182.74*	4.48E+08*	175.39*	− 0.0883*	253.47*
(C2)	1st	321.95	89.63	7.68E+12	86.95	0.0011	86.59
			94.92*	5.78E+13*	92.24*	0.0179*	86.48*
	2nd	395.01	9.10	1.79E−02	5.82	− 0.2807	116.69
			15.67*	2.27E−01*	12.39*	− 0.2596*	114.93*
	3rd	553.19	62.82	8.33E+03	58.23	− 0.1750	155.03
			71.39*	5.96E+04*	66.79*	− 0.1586*	154.54*
	4th	627.71	216.48	1.54E+16	211.26	0.0588	174.37
			230.36*	2.31E+17*	225.14*	0.0813*	174.10*
	5th	713.78	192.97	2.26E+12	187.03	− 0.02	198.21
			204.72*	1.67E+13*	198.78*	0.0010*	198.09*
(C3)	1st	384.39	94.76	2.81E+11	91.56	− 0.0278	102.27
			100.77*	1.93E+12*	97.57*	− 0.0118*	102.12*
	2nd	633.55	184.68	4.38E+13	179.41	0.0100	173.09
			193.47*	2.41E+14*	188.22*	0.0242*	172.94*
	3rd	822.38	181.37	5.34E+09	174.54	− 0.0671	229.74
			195.51*	4.39E+10*	188.67*	− 0.0496*	229.47*
(C4)	1st	508.19	25.51	6.24E−01	21.29	− 0.2533	150.00
			34.44*	6.82E+00*	30.22*	− 0.2334*	148.82*
	2nd	635.70	173.34	1.80E+12	168.06	− 0.0166	178.62
			184.25*	1.48E+13*	178.96*	0.0009*	178.40*
	3rd	693.05	219.40	1.14E+15	213.64	0.0363	188.47
			226.70*	4.04E+15*	220.94*	0.0468*	188.47*

*Values evaluated by Horowitz-Metzger equation.

### Molecular modeling

#### Geometry optimization

**Fig. 9:**
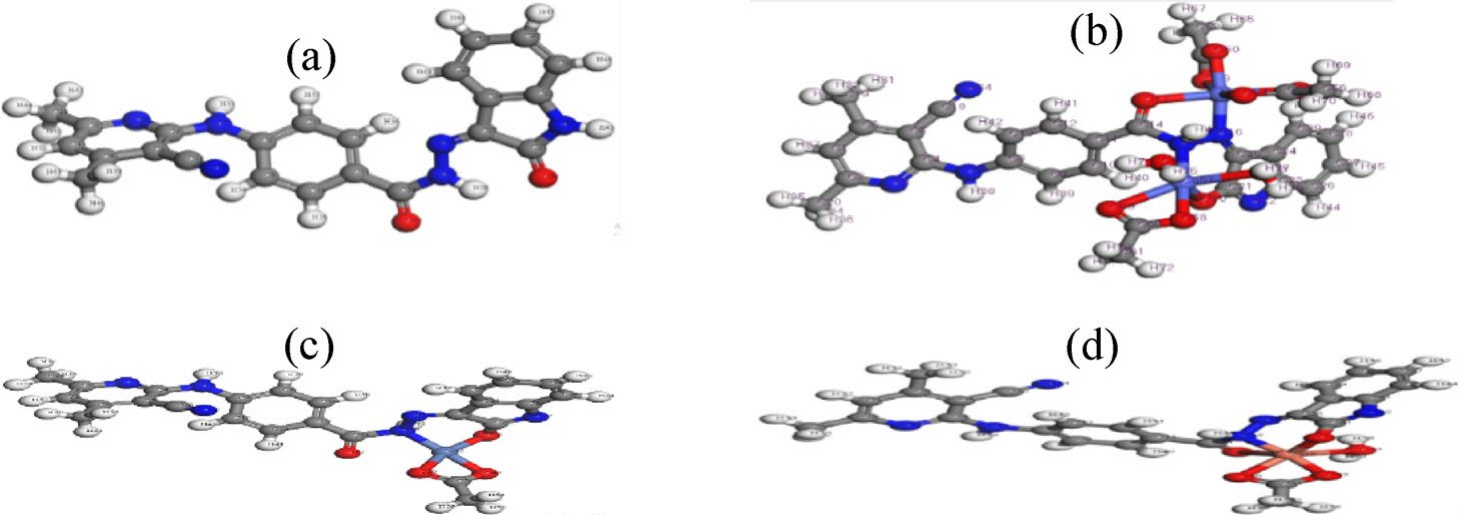
Molecular modelling with atom numbering of (**a**) H_2_BISH, (**b**) [Co_2_(HBISH)(CH_3_COO)_3_(H_2_O)_2_]·H_2_O, (**c**) [Ni(H_2_BISH)(CH_3_COO)]·2.5H_2_O and (**d**) [Cu(HBISH)(CH_3_COO)(H_2_O)].

The molecular structures of H_2_BISH (C1) and its metal complexes are illustrated in Fig. 9 and data including the bond lengths and bond angles of all compounds (Table 7).

(i) In [Co_2_(HBISH)(CH_3_COO)_3_(H_2_O)_2_]·H_2_O(C2), [Ni(H_2_BISH)(CH_3_COO)]0.2·5H_2_O(C3) and [Cu(HBISH)(CH_3_COO)(H_2_O)](C4), the bond length of N(15)-N(16) becomes longer upon coordination through the nitrogen atoms in (2), (3) and (4) complexes^50^. Also, the bond length C(14)–N(15) elongated in all complexes except complex (C4) ^50^.
(ii) In all complexes; C(21)–O(30) bond length of the isatin moiety become longer due to coordination through its oxygen atom forming M–O bond.
(iii) The H_2_BISH ligand bond angles have quite changed after coordination^50^, but the angles surrounding the metal core have big differences. The largest change occur at C(11)–C(14)–O(17), O(17)–C(14)–N(15), C(11)–C(14)–N(15), C(14)–N(15)–N(16), N(15)–N(16)–C(25), N(16)–C(25)–C(21) and O(30)–C(21)–C(25) complexes angles which increased or decreased than the ligand values.
(iv) All bond angles in [Co_2_(HBISH)(CH_3_COO)_3_(H_2_O)_2_]·H_2_O and [Cu(HBISH)(CH_3_COO)(H_2_O)] complexes agree with *SP*^3^*d*^2^ or *d*^2^*sp*^3^ hybridization of the octahedral geometry and [Ni(H_2_BISH)(CH_3_COO)]0.2·5H_2_O complex agree with sp^3^ the hybridization of tetrahedral geometry ^50^.

**Table 7:** The selected bond lengths (Å) and bond angles (°) of the H_2_BISH ligand and its metal complexes using DMOL^3^ calculations.

	Compound			
	(C1)	(C2)	(C3)	(C4)
Bond				
C(14)–O(17)	1.248	1.248	1.439	1.258
C(14)–N(15)	1.401	1.511	1.492	1.398
N(15)–N(16)	1.344	1.554	1.474	1.357
N(16)–C(25)	1.306	1.311	1.459	1.303
C(25)–C(21)	1.510	1.536	1.523	1.528
C(21)–O(30)	1.249	1.302	1.454	1.273
C(21)–N(22)	1.377	1.330	1.476	1.349
N(15)–H(38)	1.027	1.036	1.088	1.018
M–O(17)	–	2.234	–	2.359
M–N(15)	–	1.981	2.044	–
M–N(16)	–	1.894	–	2.047
M–O(30)	–	1.914	2.054	2.224
Angle				
C(10)–C(11)–C(14)	117.047	123.830	122.103	124.750
C(11)–C(14)–O(17)	123.224	122.922	119.840	122.188
O(17)–C(14)–N(15)	116.118	115.055	117.272	119.348
C(11)–C(14)–N(15)	120.658	121.933	122.534	118.219
C(14)–N(15)–N(16)	123.886	102.554	110.803	117.061
N(15)–N(16)–C(25)	119.154	115.400	117.007	125.035
N(16)–C(25)–C(21)	128.531	128.995	128.207	114.728
O(30)–C(21)–C(25)	128.021	126.038	125.177	122.000
O(30)–C(21)–N(22)	126.638	123.060	123.653	127.510
M–O(30)–C(21)	–	122.292	108.821	107.154
N(15)–M–O(30)	–	92.539	92.022	–
O(17)–M–N(16)	–	81.524	–	73.151
N(16)–Cu–O(30)	–	–	–	80.045
M–N(15)–N(16)	–	–	112.981	–

#### Global reactivity descriptors

**Fig. 10:**
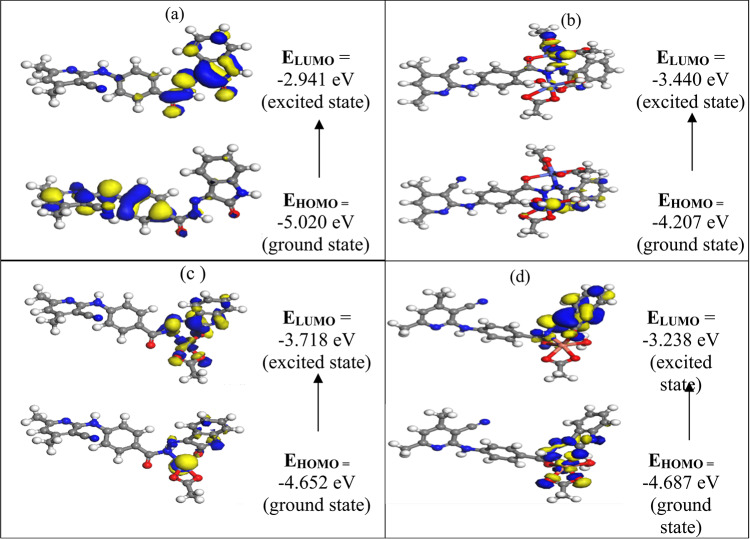
Frontier orbitals (HOMO & LUMO) of (**a**) H_2_BISH, (**b**) [Co_2_(HBISH)(CH_3_COO)_3_(H_2_O)_2_]·H_2_O, (**c**) [Ni(H_2_BISH)(CH_3_COO)]·2.5H_2_O and (**d**) [Cu(HBISH)(CH_3_COO)(H_2_O)].

The stability of all investigated compounds is revealed by their negative values of the energies of all frontier molecular orbitals viz E_HOMO_ (highest occupied molecular orbitals) and E_LUMO (_lowest unoccupied molecular orbitals) that are important parameter in quantum calculations (Table 8 and Fig. 12). The energy band gap, ∆E_g_ = E_LUMO_ − E_HOMO_ is a predication of capability of a compound for charge transfer (CT). The small energy gap value of H_2_BISH reveals the ease of CT which reveal in turn the higher polarizability, increased reactivity, the softness of the molecule and higher electron donating ability (Fig. 10)^51^.

- The HOMO is localized on carbonyl O(17), N (15), N (16), and O (30), indicating that the mentioned atoms are the most probable targets due to the nucleophile attack at the metal ion’s center^51^.
- A glance at Table 8 indicates that the energy band gap E_g_ values of title compounds are small lying in the range of semiconductors following the sequence: (Complex (C2) (0.767 eV) > Complex (C3) (0.934 eV) > Complex (C4)(1.449 eV) > ligand (C1) (2.079 eV)^51^ which are in accordance with values of semiconductors (0.7–4.45 eV) reported in literature^52^ suggesting the prepared substances to be promising ones for harvesting solar radiation in solar cell applications.

**Table 8:** Calculated E_HOMO_, E_LUMO_, energy band gap (E_H_–E_L_), electronegativity (χ), chemical potentials (Pi), absolute hardness (*η*), absolute softness (*S*), global electrophilicity index (ω) and electronic charge (∆N_max_) for H_2_BISH and its metal complexes (C1–C4).

Compound	HOMO	LUMO	E_H_–E_L_	X	Pi	η	S	ω	σ	∆N_max_
(C1)	− 5.020	− 2.941	− 2.079	3.981	− 3.981	1.040	0.481	7.621	0.962	3.829
(C2)	− 4.207	− 3.440	− 0.767	3.824	− 3.824	0.384	1.304	19.060	2.607	9.970
(C3)	− 4.652	− 3.718	− 0.934	4.185	− 4.185	0.467	1.071	18.752	2.141	8.961
(C4)	− 4.687	− 3.238	− 1.449	3.963	− 3.963	0.725	0.690	10.836	1.380	5.469

The other global reactivity descriptors such as the electronegativity (*χ*), chemical potentials (Pi), absolute hardness (*η*), absolute softness (*S*), global electrophilicity index (*ω*) and electronic charge (∆N_max_) are calculated also as previous^52^ and reported in Table 8.

#### Molecular electrostatic potential (MEP) surfaces

**Fig. 11:**
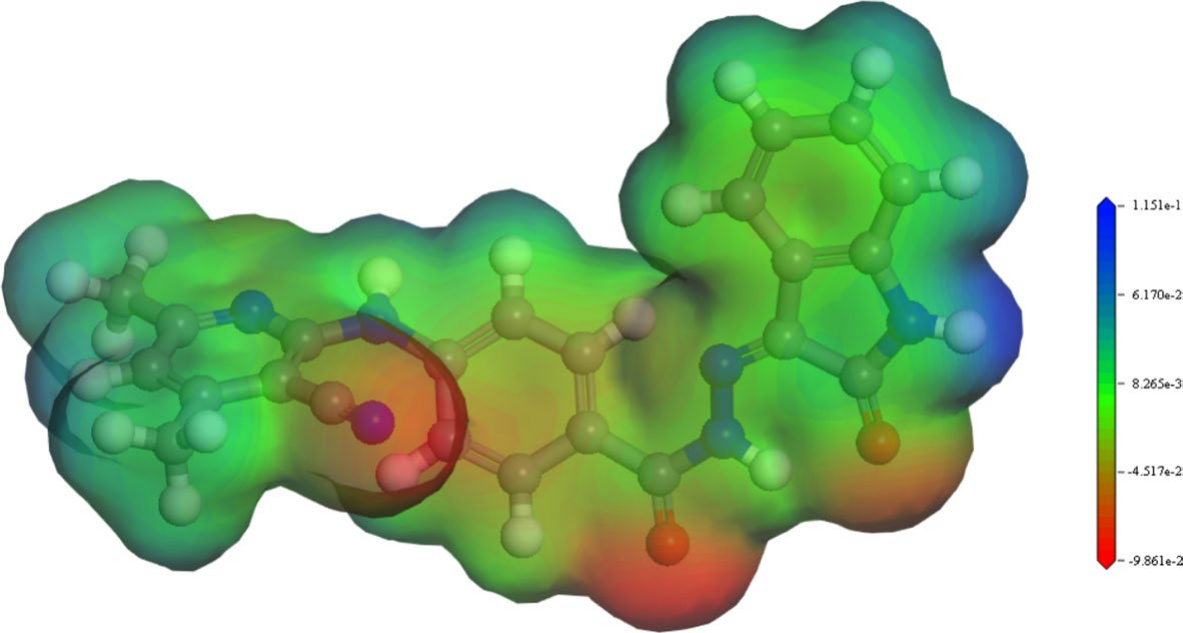
The MEP surface of H_2_BISH ligand.

Figure 11 indicates MEP of H_2_BISH 3D-charge distribution surfaces^53^. It helps to illustrate the ligand relative polarity where the positive and negative charged regions are represented with blue and red colors. Red preferred the site for electrophilic attack and blue positive zone for nucleophilic attack. The potential follows the order: Blue > green > red. As can be seen from the designated molecule’s MEP map, the electronegative nitrogen atoms have negative potential and the hydrogen atoms have positive potential. MEP is an important tool when studying the relationship between a drug or biomolecule molecular structure and its physiochemical properties^54^.

### Some biological studies

#### Antibacterial

**Fig. 12:**
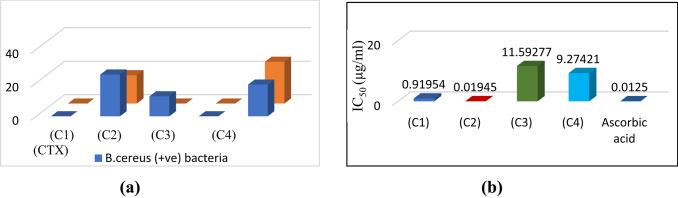
Antibacterial activity of ligand (H_2_BISH) (C1) and its metal complexes (C2–C4).

The antibacterial efficacy data (Fig. 12a and Table 9) against Gram-positive (*B. cereus*) and Gram-negative (*E. coli*) bacteria were tested for H_2_BISH (C1) and its metal complexes (C2–C4). The data demonstrated no activity of the parent hydrazone (C1) and Cu(II) complex (C4) against both bacterial strains. On the other hand, the binuclear Co(II) (C2) showed an inhibition power (inhibition zone diameter = 25 mm) exceeded the antibiotic itself (inhibition zone diameter = 19 mm) towards the growth of Gram-positive (*B. cereus*) and moderate activity (~ % 68) towards the growth of *E. coli* bacterium. Ni(II) (C3) complex showed a response only towards Gram-positive (*B. cereus*) (%63 inhibition activity). The higher activity of binuclear Co (II) complex (C2) may be referred to the existence of two metal ions and three acetate molecules that enhance the lipophilicity and thus facilitate the penetration of the complex through the bacterium membrane. i.e. in turn causes damage of DNA in the cell and consequently killing the organism. Generally, the results revealed higher activity upon chelation according to Overtone theory^55^ where the complexes nature are more lipophilic. Complexation decreases the metal ion polarity as it appends + ve charge on the ligand donor atoms in addition to the π-electron cloud delocalization surrounding the chelating ring. The metal atom lipophilicity permits it more effectively to penetrate the bacteria lipid membranes so inhibit the division of the cell. In addition, there are also various parameters affecting the activity against the bacterial cell. Firstly, the nature of bacterial cell membrane (differences in cell wall structure), Gram-positive bacteria possess a thick cell wall containing many layers of peptidoglycan and teichoic acids, but in contrast Gram negative bacteria have a relatively thin cell wall consisting of a few layers of peptidoglycan can surround by a second lipid membrane containing lipopoly-saccharides and lipoproteins. The other factors controlling the antibacterial activity are the ligand structure, the metal ion size, coordination mode, complex geometry and hydrophilicity. All of this influences the activity. The values agree with the previous studies^55^.

**Table 9:** Antibacterial activity expressed in inhibition zone (mm), DPPH scavenging activity and the cytotoxic activity of H_2_BISH (C1) and its complexes (C2–C4).

Compound	Antibacterial activity (in mm)		DPPH % Scavenging activity				Cytotoxic activity
	*B. cereus* (+ ve) bacteria	*E. coli* (− ve) bacteria	IC_50_ (mg/mL)	Conc. (mg/mL)	% Inhibition	% Remaining DPPH	IC_50_
(C1)	− ve	− ve	0.82385	0.10417	62.600	37.400	157.01
				0.05208	62.893	37.107	
(C2)	25	17	0.01945	0.01300	53.981	46.019	106.00
				0.0070	53.543	46.457	
(C3)	12	− ve	11.59277	0.0880	88.532	11.468	26.23
				0.044	50.621	49.379	
(C4)	− ve	− ve	9.27421	0.1052	60.482	39.518	59.51
				0.0526	60.336	39.664	
*Cefotaxime	19	25	–	–	–	–	–
**Ascorbic acid			0.0125	–	–	–	–
Cis-platin							3.68

#### Antioxidant activity (DPPH scavenging method)

In vitro antioxidant tests using free radical scavenger, DPPH, are quite simple and easy when compared to other test methods. The assay was carried out for H_2_BISH (C1) and the complexes (C2–C4), the decreases in DPPH molar absorbance at 517 nm are recorded. Figure 12b and Table 9 illustrate the antioxidant activity of title compounds values comparable to ascorbic acid (Vitamin C) as a standard substance. According to the results obtained, the following order is observed: C2 > C1 > C4 > C3. It is clear that the complex C2 viz Co(II) complex showed IC_50_ (0.01945) near to that of the standard (IC_50_ = 0.01250) activity and higher than the parent hydrazone, C1 or other metal complexes viz C3 and C4. This may be referred to the structure of the Co(II) complex as it contains two metal ions, three acetate ions that contain extra donating groups (3 C=O) in addition to 3O^−2^ that can donate an electron enhancing the free radical scavenging ability. This finding is consistent with the E_g_ value (− 0.767 eV) obtained for the Co(II) complex which is very small suggesting higher polarizability, increased reactivity, the softness of the molecule and higher electron donating ability. An insight to Table 9 shows clearly that, the compounds under investigation possess great activities in general when compared with the ascorbic acid, in turn these compounds can be promising candidate in antioxidant drug field^56^.

#### In vitro cytotoxic activity (MTT assay)

**Fig. 13:**
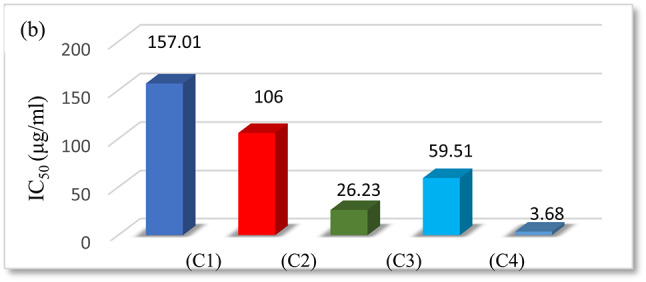
The IC_50_ values of the ligand and its metal complexes of in vitro antitumor activity against MCF7.

A colorimetric assay (MTT assay), which measures mitochondrial dehydrogenase activity as an indicator of cell viability, was used to conduct in vitro cytotoxicity tests using all of the synthesized compounds against the human cancer cell line (MCF7) normal cell line. Simultaneously, cisplatin, a widely utilized anticancer treatment, has also been considered as a standard. The graph that shows the percentage of cells that are viable and the concentration was used to determine the IC_50_ values (Fig. 13 and Table 9). The IC_50_ values for all substances ranged from 26.23 to 157.01 μg/mL, suggesting that they all had anticancer activity against the cell lines without seriously harming the healthy cells. Comparing the ligand and its complexes cytotoxicity activity shows the Ni(II) acetate (C3) complex exhibited high activity. This indicates that the complicated biological activity of compounds must be expressed through their chemical structure, which can also play a significant role in the discovery and manufacture of new chemotherapy drugs^57^.

#### Molecular docking

**Fig. 14:**
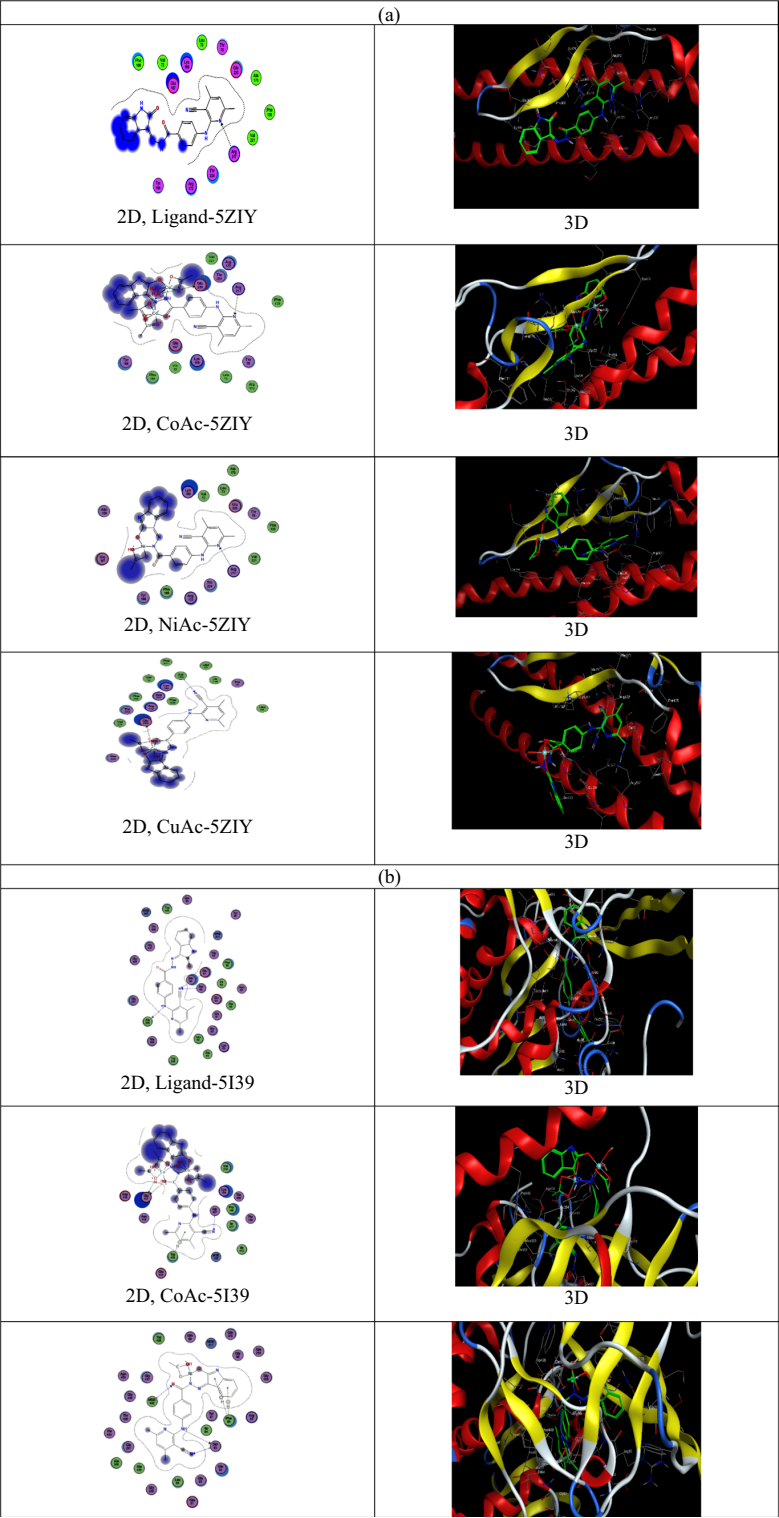

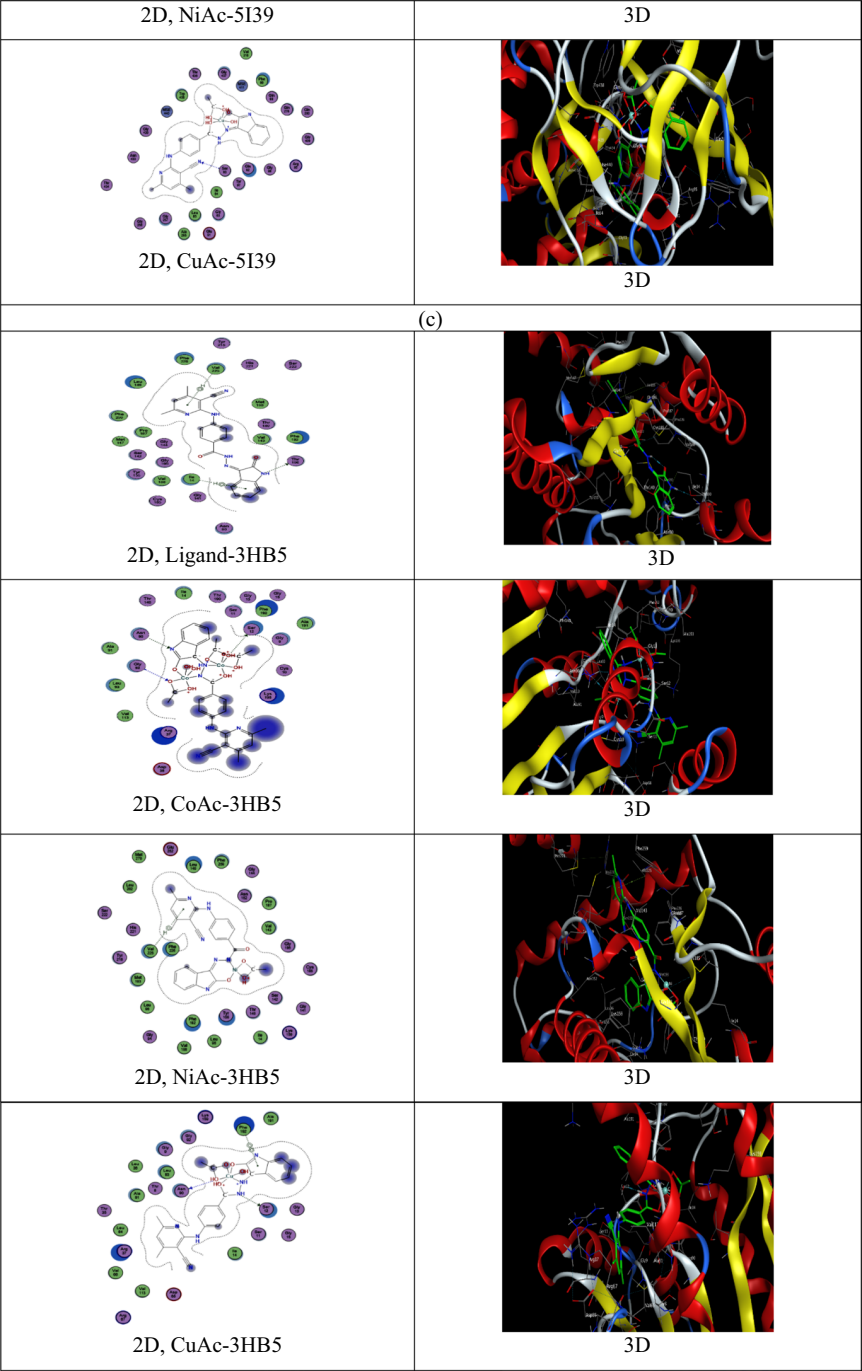
Two-dimensional and three-dimensional plots of the interaction between the ligand (C1) and its metal complexes (C2–C4) with the active site of the receptor of *Bacillus cereus* (PDB ID: 5ZIY) (**a**), *E. coli* (PDB ID: 5I39) (**b**), and *Breast cancer* (PDB ID: 3HB5)(**c**).

Molecular docking is very essential tool for the development of computer drugs. Determining the similar binding modes of *Bacillus cereus* (PDB ID: 5ZIY), *E. coli* (PDB ID: 5I39) and *Breast cancer* (PDB ID: 3HB5) were undergo with The MOE2022 program. The 2D and 3D docking plots images of the ligand (C1) and its complexes (C2–C4) with *Bacillus cereus, E. coli* bacteria and *Breast cancer* were illustrated in Fig. 14a–c. Figure 14 was investigated the 2D Map of the binding sites in ligand–protein complexes for the studied compounds observed the formed hydrogen bonds with dashed lines. This docking study is like the actual docking cycle, and Table 10 reports the ligand and proteins energy interaction. The ligand–protein binding surfaces and its complexes are found particularly consisting of hydrogen donor and acceptor, pi–pi, pi–H and H–pi bonds. Remembering the fact when the negative binding affinity greater than (− 5.0) refers to that it’s highly likely to happen. The ligand links with the proteins of the *Bacillus cereus*, *E. coli,* and *Breast cancer* by pi–hydrogen and hydrogen donor and acceptor. The bonds between the free ligand and the amino acid show the following distances: 3.45 for *Bacillus cereus,* 3.30–3.49 Å for *E. coli*, and 3.26–4.35 for *Breast cancer*. The moderate binding energy values of unbound ligands (S = − 7.14 to − 5.24 kcal/mol) are notable for 5ZIY, (S = − 10.10 to − 5.88 for *E. Coli*, and S = − 8.71 to − 5.74 kcal/mol for 3HB5. The most common technique to verify the computed binding affinity of the prepared compounds uses the compounds binding energies. The binding energy of the compound (ligand) to the receptor was decreased by the mutation which increased their propensity to link the receptor. One feature of the ligand (C1) and its complexes (C2–C4) is the availability of numerous open, active hydrogen bonding sites. This research thankfully shows, these compounds (C1–C2) can now actively block protein binding which also helps to produce other inhibitory compounds. The results clearly observe that the ligand (C1) and its complexes (C2–C4) succussed to inhibit the *Bacillus cereus* (PDB ID: 5ZIY) (Fig. 14a), *E. coli* (PDB ID: 5I39) (Fig. 14b), and *Breast cancer* (PDB ID: 3HB5) (Fig. 14c),. for ligand (C1), in the order, 5I39 (four bonds) > 3HB5 (three bonds) > 5ZIY (one bond), for Co-complex (C2), the order is 5I39 (5 bonds) > 3HB5 (3 bonds) > 5ZIY (two bonds), for Ni-complex (C3), the order is 5I39 (5 bonds) > 5ZIY (one bond) = 3HB5 (one bond), for Cu-complex (C4), 3HB5 (three bonds > 5ZIY (2 bonds) > 5I39 (one bond). These great results support the possibility of manufacturing a novel promising drugs for *Bacillus cereus*, *E. coli* and *Breast cancer*.

**Table 10:** Data of the docking interaction calculations between the ligand (C1) and its complexes (C2–C4) with the *Bacillus cereus* receptor’s active site (PDB ID: 5ZIY), *E. Coli* (PBD ID: 5I39) and *Breast cancer* (PDB: 3HB5).

System	Binding score (kcal/mol)	Receptor	Interaction	Distance (Å)	E (kcal/mol)
Ligand (C1)-*Bacillus cereus* (5ZIY)					
N 4	− 5.52	CD ARG 217	H-acceptor	3.45	− 0.8
Co-complex (C2)-5ZIY					
O 63	− 5.71	OE2 GLU 220	H-donor	2.67	− 4.5
N 4		CD ARG 217	H-acceptor	3.46	− 0.7
Ni-complex (C3)-5ZIY					
N 4	− 6.14	CD ARG 217	H-acceptor	3.44	− 0.8
Cu-complex (C4)-5ZIY					
O 54	− 5.74	OE2 GLU 220	H-donor	2.57	− 11.0
N 61		CA ALA 170	H-acceptor	3.28	− 1.0
Ligand (C1)-*E. Coli* (5I39)					
N 8		O ALA 255	H-donor	3.49	− 0.8
N 49	− 8.69	N GLU 91	H-acceptor	3.43	− 1.8
N 49		N SER 93	H-acceptor	3.34	− 2.7
N 49		CB SER 93	H-acceptor	3.30	− 0.5
Co-complex (C2)-5I39					
O 24		OE1 GLU 417	H-donor	2.79	− 13.8
O 24	− 5.88	OE2 GLU 417	H-donor	3.23	− 3.9
O 57		OE1 GLU 417	H-donor	2.56	− 7.8
N 79		N GLY 437	H-acceptor	3.17	− 1.7
6-ring		NE1 TRP 438	pi–H	3.62	− 0.8
Ni-complex (C3)-5I39					
N 8		OG SER 93	H-donor	2.82	− 1.6
O 23	− 9.77	N MSE 440	H-acceptor	3.30	− 1.9
N 56		N SER 93	H-acceptor	3.10	− 4.1
5-ring		CA PHE 96	pi–H	4.22	− 0.7
6-ring		CA PHE 96	pi–H	3.93	− 0.9
Cu-complex (C4)-5I39					
N 61	− 7.24	N SER 93	H-acceptor	2.77	− 4.7
Ligand (C1)-*Breast cancer* (3HB5)					
N 34	− 8.35	OG1 THR 190	H-donor	3.26	− 1.1
6-ring		CD1 ILE 14	pi–H	3.65	− 0.6
6-ring		CG1 VAL 225	pi–H	4.35	− 0.5
Co-complex (C2)-3HB5					
O 64	− 5.74	OG SER 12	H-donor	2.82	− 0.9
N 35		ND2 ASN 90	H-acceptor	3.33	− 0.5
O 72		N GLY 92	H-acceptor	3.07	− 2.5
Ni-complex (C3)-3HB5					
6-ring	− 8.19	CG1 VAL 225	pi–H	3.99	− 0.9
Cu-complex (C4)-3HB5					
N 21	− 7.14	OG SER 12	H-donor	2.97	− 3.3
O 54		O ASN 90	H-donor	2.98	− 8.9
5-ring		6-ring PHE 192	pi–pi	3.95	− 0.0

## Conclusion

A binuclear Co(II) (C2) and mononuclear Ni(II) (C3) and Cu(II) (C4) acetate complexes of a novel Schiff base namely, (*Z*)-4-((3-cyano-4,6-dimethylpyridin-2-yl)amino)-*N*′-(2-oxoindolin-3-ylidene)benzohydrazide (H_2_BISH)(C1) were synthesized and characterized. Ni(II) complex (C3) has adopted a tetrahedral geometry while cobalt (II) (C2) and copper (II) (C4) assigned an octahedral one. The structure of cobalt complex (C2) was further studied by XRD indicating indicating a hexagonal C_12_ H_54_ Cl Co_2_ N_12_ O_38_ V_15_ geometry with lattice constants a = b = 12.1200 Å; c = 22.0530 Å and P 63/m m c space group. ESR spectrum of C4 exhibited a broad single line concentrated at g_iso_ = 2.06 accompanying with characterized hyperfine structure, indicating that the hydrazone (ONO) chelated system observes to be coplanar with two chelating rings which agree with the planar octahedral geometry (g_||_ (2.290)  >  g_⊥_ (2.068) > 2.0023) referring that the copper core’s *d*_*x*_^2^*–*_*y*_^2^ ground-state. TGA and DrTGA analysis indicated increasing of thermal stability of parent hydrazone upon complexation and thermal degradation is a nonspontaneous (= Ve ΔG^*^), endothermic process (+ ve ΔH^*^) and low internal energy of title compounds as revealed by (− ve ΔS^*^) values. The energy band gap, ∆E_g_ = E_LUMO_ − E_HOMO_ calculated by DFT of is a predication of capability of a compound for charge transfer (CT). All title compounds have small negative E_g_ of order: C2 < C3 < C4 < C1 (H_2_BISH) revealing the ease of CT in turn the higher polarizability, increased reactivity and softness as well as the values lie in the range of semiconductor suggesting the possibility of utilization of present compounds in solar cells. Also, the compounds were screened for antibacterial and breast anticancer activities. Only C2 and C3 showed inhibition power; (C2): a higher and a moderate activity towards *B. cereus* (Gram + ve) and *E. coli* (Gram − ve), respective comparable to the standard drug (*Cefotaxim*) while (C3) showed good inhibition efficiency against *B. cereus* only. With respect to in vitro cytotoxic activity (MTT assay) against human cancer cell line (MCF7), the IC_50_ values for all compounds ranged from 26.23 to 157.01 μg/mL, suggesting that they all possess anticancer efficiency. Among all compounds, tetrahedral Ni(II) acetate (C3) complex exhibited the potent activity revealing that the complicated biological activity of compounds must be expressed through their chemical structure. Molecular docking was done for determining the similar binding modes of *the protein of Bacillus cereus* (PDB ID: 5ZIY), *E. coli* (PDB ID: 5I39) and *Breast cancer* (PDB ID: 3HB5) were undergo with The MOE2022 program. The data indicated that the ligand–protein binding surfaces and its complexes are found particularly consisting of hydrogen donor and acceptor, pi–pi, pi–H and H–pi bonds.

## Supplementary Information

Below is the link to the electronic supplementary material.

Supplementary Material 1

## Data Availability

All the data generated or used in the study is provided in the manuscript.
