# Supplementary material for: Synthesis, computational, biological activity and molecular docking study of Co2+, Ni2+ and Cu2+ chelates of a new indolbenzohydrazone NO donor
Source: Sci Rep. 2025 Oct 28;15:37673. doi: 10.1038/s41598-025-20172-w (PMC12569096; doi:10.1038/s41598-025-20172-w)
Supplement: Supplementary file 1 — Supplementary Material 1 [file 41598_2025_20172_MOESM1_ESM.docx]

**Supplementary material**

**Table 1S**. Physical properties and elemental analyses of H_3_BISH and its metal complexes.

| **Compound**  **(Mol. Wt.)** | **Color** | **M.P.**  **^o^C** | **Found (Calcd.) %** | | | | | **Yield**  **%** |
| --- | --- | --- | --- | --- | --- | --- | --- | --- |
|  |  |  | **C** | **H** | **N** | **Cl** | **M** |  |
| H_3_BISH **(1)**  C_23_H_18_N_6_O_2_ (410.44) | Dark yellow | 290 | 65.59  (67.31) | 4.42  (4.21) | 20.04  (20.48) | - | - | 91 |
| [Co(H_2_BISH)Cl(H_2_O)].0.5H_2_O **(2)** C_23_H_20_ClCoN_6_O_3.5_ (530.84) | Pale yellow | >300 | 51.46  (52.04) | 3.53  (3.80) | 15.45  (15.83) | 6.36  (6.68) | 10.73  (11.10) | 92 |
| [Co_2_(H_2_BISH)(CH_3_COO)_3_(H_2_O)_2_].H_2_O **(3)** C_29_H_32_Co_2_N_6_O_11_ (758.48) | Reddish brown | >300 | 45.08  (45.92) | 4.11  (4.25) | 11.32  (11.08) | - | 15.28  (15.54) | 95 |
| [Cu(H_3_BISH)Cl_2_(H_2_O)_2_] **(4)**  C_23_H_22_Cl_2_CuN_6_O_4_ (580.92) | Reddish brown | >300 | 47.83  (47.55) | 3.51  (3.82) | 13.97  (14.47) | 12.64  (12.20) | 10.62  (10.94) | 79 |
| [Cu(H_3_BISH)(CH_3_COO)(H_2_O)] **(5)**  C_25_H_22_CuN_6_O_5_ (550.04) | Reddish brown | >300 | 55.10  (54.59) | 4.24  (4.03) | 16.14 (15.28) | - | 11.93  (11.55) | 71 |

**3.7. Thermal analysis**

**3.7.1. Thermogravimetric studies**

|  |  |
| --- | --- |
|  |  |

**Figure 1S: Thermal analysis curves (TGA, DTG) of (a) H_2_BISH, (b) [Co_2_(HBISH)(CH_3_COO)_3_(H_2_O)_2_].H_2_O, (c) [Ni(H_2_BISH)(CH_3_COO)].2.5H_2_O and (d) [Cu(HBISH)(CH_3_COO)(H_2_O)].**

|  |  |
| --- | --- |
|  |  |

**Figure 2S: Horowitz-Metzger plots of the first degradation step for (a) H_2_BISH, (b) [Co_2_(HBISH)(CH_3_COO)_3_(H_2_O)_2_].H_2_O, (c) [Ni(H_2_BISH)(CH_3_COO)].2.5H_2_O and (d) [Cu(HBISH)(CH_3_COO)(H_2_O)].**
